# Decoding Task-Specific Cognitive States with Slow, Directed Functional Networks in the Human Brain

**DOI:** 10.1523/ENEURO.0512-19.2019

**Published:** 2020-07-07

**Authors:** Shagun Ajmera, Hritik Jain, Mali Sundaresan, Devarajan Sridharan

**Affiliations:** 1 Centre for Neuroscience, Indian Institute of Science, Bangalore 560012, India; 2 Department of Computer Science and Automation, Indian Institute of Science, Bangalore 560012, India

**Keywords:** cognitive score prediction, emergent dynamics, functional connectivity, Granger causality, partial correlations, support vector machines

## Abstract

Flexible functional interactions among brain regions mediate critical cognitive functions. Such interactions can be measured using functional magnetic resonance imaging (fMRI) data either with instantaneous (zero-lag) or lag-based (time-lagged) functional connectivity. Because the fMRI hemodynamic response is slow, and is sampled at a timescale (seconds) several orders of magnitude slower than the underlying neural dynamics (milliseconds), simulation studies have shown that lag-based fMRI functional connectivity, measured with approaches like Granger–Geweke causality (GC), provides spurious and unreliable estimates of underlying neural interactions. Experimental verification of this claim is challenging because neural ground truth connectivity is often unavailable concurrently with fMRI recordings. Here we demonstrate that, despite these widely held caveats, GC networks estimated from fMRI recordings contain useful information for classifying task-specific cognitive states. We estimated instantaneous and lag-based GC functional connectivity networks using fMRI data from 1000 participants (Human Connectome Project database). A linear classifier, trained on either instantaneous or lag-based GC, reliably discriminated among seven different task and resting brain states, with >80% cross-validation accuracy. With network simulations, we demonstrate that instantaneous and lag-based GC exploited interactions at fast and slow timescales, respectively, to achieve robust classification. With human fMRI data, instantaneous and lag-based GC identified complementary, task–core networks. Finally, variations in GC connectivity explained inter-individual variations in a variety of cognitive scores. Our findings show that instantaneous and lag-based methods reveal complementary aspects of functional connectivity in the brain, and suggest that slow, directed functional interactions, estimated with fMRI, may provide useful markers of behaviorally relevant cognitive states.

## Significance Statement

Functional MRI (fMRI) is a leading noninvasive technique for mapping functionally connected networks in the human brain. The fMRI hemodynamic response is slow and noisy, and is sampled far more slowly (seconds) than the timescale of neuronal spikes (milliseconds). fMRI data are, therefore, considered unsuitable for mapping directed, time-lagged functional connectivity among brain regions. Here, we apply machine learning to fMRI data from 1000 human participants and show that directed connectivity, estimated with Granger–Geweke causality from fMRI data, accurately predicts task-specific cognitive states, and individual subjects’ behavioral scores. Moreover, directed connectivity robustly identifies network configurations that may be challenging to identify with conventional correlation-based approaches. Directed functional connectivity, as measured with fMRI, may be relevant for a complete understanding of brain function.

## Introduction

Mapping functional coupling among brain regions is key to mapping brain function and for understanding how the brain produces behavior (Fox et al., 2005). Human functional magnetic resonance imaging (fMRI) studies have commonly investigated such functional coupling with correlation-based measures, including the Pearson correlation coefficient (Vincent et al., 2008; Buckner et al., 2009) and partial correlations (PCs) between pairs of brain regions (Marrelec et al., 2006; Ryali et al., 2012). Correlation-based measures characterize “instantaneous” functional interactions among brain regions that occur at timescales faster than the sampling rate of the measurement (Barnett and Seth, 2017). In contrast, comparatively few studies have characterized functional connectivity with lag-based measures (Sridharan et al., 2008; Ryali et al., 2011).

Measures of linear dependence and feedback, based on Granger–Geweke causality (GC; Geweke, 1982, 1984) represent a powerful approach for estimating both instantaneous and lag-based functional connectivity. These measures are firmly grounded in information theory and statistical inferential frameworks (Geweke, 1982, 1984; Seth et al., 2015). GC measures have been widely applied to estimate functional connectivity in recordings of brain activity made with electroencephalography (EEG; Dhamala et al., 2008), magnetoencephalography (Ding and Wang, 2014), and electrocorticography (Bastos et al., 2015). However, the application of GC measures to brain recordings made with fMRI has provoked significant controversy (Chang et al., 2008; Smith et al., 2011; Friston et al., 2013; Wen et al., 2013). Because the hemodynamic response is produced and sampled at a timescale (seconds) several orders of magnitude slower than the underlying neural processes (milliseconds), previous studies have argued that lag-based measures, particularly lag-based GC, produce spurious and unreliable estimates of functional connectivity, when applied to fMRI data (fMRI-GC; Lin et al., 2009; Smith et al., 2011; Seth et al., 2013; Solo et al., 2018).

Three primary confounds have been identified with inferring connectivity with fMRI-GC. First, systematic differences in hemodynamic lags across regions could yield spurious directionality of GC connections (Chang et al., 2008; Friston, 2009; Smith et al., 2011). Second, in simulations, measurement noise added to the signal during fMRI acquisition significantly degrades GC functional connectivity estimates (Nolte et al., 2008; Smith et al., 2012; Seth et al., 2013). Finally, downsampling recordings to the typical fMRI sampling rate (seconds), three orders of magnitude slower than the timescale of neural spiking (milliseconds), effectively eliminates all traces of functional connectivity inferred by GC (Seth et al., 2013).

The controversy regarding the application of GC to fMRI data continues to date. On the one hand, claims regarding the efficacy of GC estimates are primarily based on simulations (Seth et al., 2015; Solo, 2016) and are only as valid as the underlying model of neural activity and hemodynamic responses. Because the precise mechanism by which neural responses generate hemodynamic responses is an active area of research, strong conclusions cannot be drawn based on fMRI simulations alone. On the other hand, establishing ground truth validity for fMRI functional connectivity requires invasive neurophysiological recordings across many brain regions, concurrently during fMRI scans, a challenging enterprise. For example, David et al. (2008) addressed this technical challenge, and showed that, in a rodent model, fMRI-GC functional connectivity estimates matched connectivity estimates from intracerebral EEG only when confounding hemodynamic effects were explicitly removed from the former.

Here, we seek to examine the empirical relevance of fMRI-GC functional connectivity networks in human subjects for identifying task-specific cognitive states, and for predicting behavior, by applying machine learning (Arbabshirani et al., 2017) to fMRI-GC networks. We estimated instantaneous GC (iGC) and lag-based GC connectivity with fMRI data drawn from 1000 human subjects, recorded under seven different task conditions and in the resting state [Human Connectome Project (HCP) database; Glasser et al., 2013]. We trained a linear classifier, based on GC connectivity features, to discriminate among the different task and resting conditions, and assessed classifier accuracy with cross-validation. Instantaneous and lag-based fMRI GC connectivity could decode task-specific cognitive states with superlative accuracies. Next, with simulations, we show that slow interactions at the timescale of seconds emerge in networks with sparse, random connectivity (Ganguli et al., 2008), despite individual neurons operating at fast, millisecond, timescales. We further show that such interactions can be recovered with GC sampled at slow fMRI timescales, providing a putative explanation for the success of GC with classifying task states (Sundaresan et al., 2017). Finally, we demonstrate that GC connectivity features can be used as predictors (Liégeois et al., 2019) to explain interindividual variations in behavioral scores across a variety of cognitive tests. In summary, fMRI-GC may be relevant for understanding slow, emergent, and behaviorally relevant functional interactions in the human brain.

## Materials and Methods

### Ethics statement

The scanning protocol for the HCP was approved by the Human Research Protection Office at Washington University at St. Louis (institutional review board #201204036). Only deidentified, publicly released data were used in this study. Secondary data analysis procedures were approved by the Institutional Human Ethics Committee at the Indian Institute of Science (Bangalore, India).

### fMRI data, parcellation, and time series extraction

We analyzed minimally preprocessed brain scans of 1000 subjects, drawn from the HCP database (S1200 release; age range, 22–35 years; 527 females); fMRI acquisition and preprocessing details have been described previously (Van Essen et al., 2012; Glasser et al., 2013). Briefly, in this preprocessing pipeline, a subject’s data are first aligned to MNI space, volumes are segmented based on predefined subcortical parcels, and white matter and pial (cortical) surfaces are registered to the respective surface atlas. This is followed by gradient distortion correction, motion correction, image distortion correction, spline resampling, intensity normalization, and brain masking. Next, cortical and subcortical gray matter voxels are mapped onto standard cortical surface vertices and subcortical parcels, respectively. Extended Data Figure 1-3 shows the identifiers of the subjects from whom data were analyzed. Data were analyzed from resting state and seven other task conditions (Extended Data Fig. 1-1), as follows: emotion processing, gambling, language, motor, relational processing, social cognition and working memory. In most figures, these tasks are referred to with their initial letters. fMRI scans for the relational task were not available for 9 of 1000 subjects; therefore, we analyzed a total of 7991 scans across all tasks and subjects.

**Figure 1.**
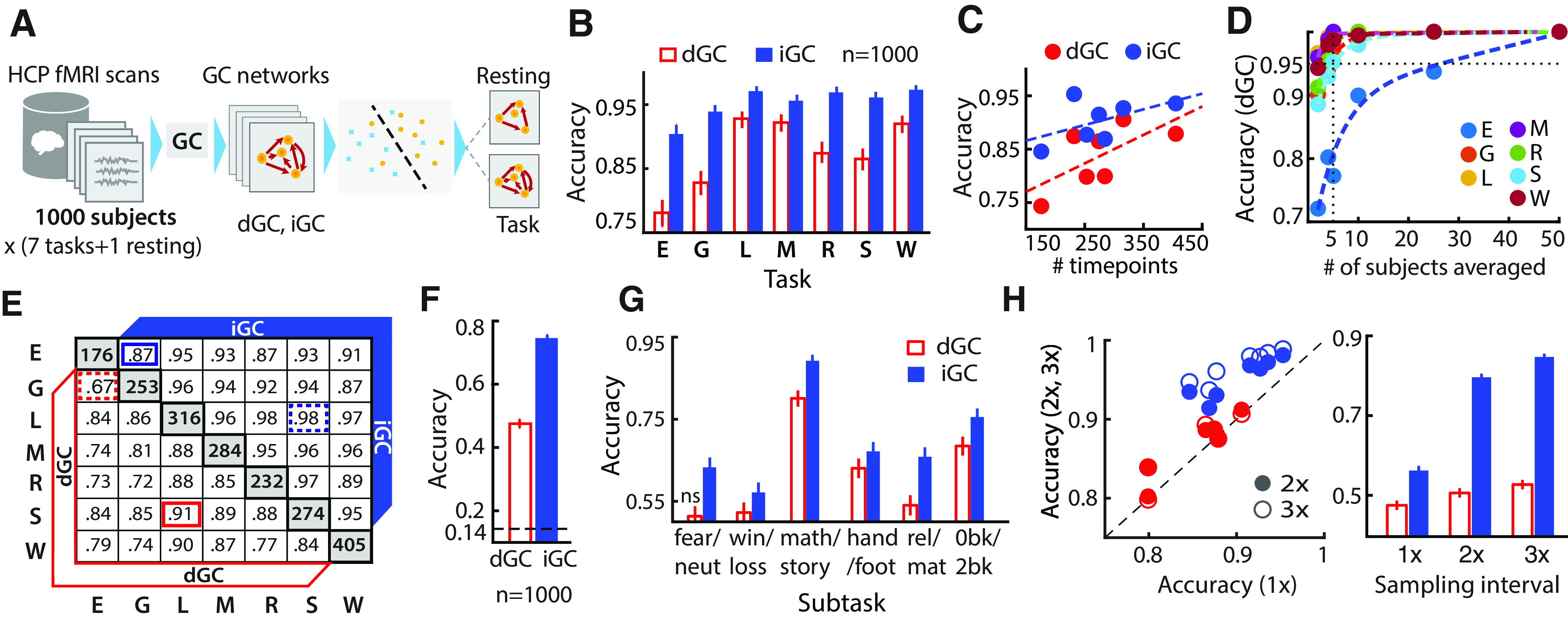
Discriminating between task and resting state with instantaneous GC and directed GC networks. ***A***, Schematic of task state classification based on iGC and dGC with fMRI data from 1000 subjects (see text for details; IDs in Extended Data Fig. 1-3). ***B***, Two-way classification accuracies (leave-one-out) for each of seven tasks versus resting state based on GC. Red unfilled bars and blue filled bars: accuracies based on dGC and iGC features, respectively (Extended Data Fig. 1-1, task key). Error-bars, Clopper–Pearson binomial confidence intervals. Chance accuracy, 0.5 (data not shown). ***C***, Two-way task versus resting-state classification accuracies based on dGC (red dots) and iGC (blue dots), as a function of the number of task scan timepoints (volumes). Dashed lines, Linear fits. ***D***, Two-way task versus resting-state classification accuracies based on dGC after averaging dGC matrices over different numbers of subjects (*x*-axis). Each task is represented with a different color. Colored dashed lines, Biexponential fits; black dashed horizontal and vertical lines, 95% accuracy and *n* = 5 subjects’ average, respectively. ***E***, Two-way classification accuracies across each pair of tasks. Cells, Classification accuracies for each pair of tasks based on dGC (lower triangular matrix) or iGC (upper triangular matrix); diagonal cells, number of task scan timepoints; highlighted cells, lowest (dashed-line border) and highest (solid-line border) accuracies achieved with dGC (red) and iGC (blue). ***F***, N-way classification accuracies among all seven tasks. Dashed line, Chance accuracy (14.3%). Other conventions are the same as in ***B***. ***G***, Two-way subtask classification accuracies (Extended Data Fig. 1-2, descriptions) based on GC. ns, Accuracy not significantly above chance. Other conventions are the same as in ***B***. ***H***, Left, Two-way task versus resting-state classification accuracies obtained with regional time series subsampled at 2× (filled symbols) and 3× (open symbols) of the TR (720 ms; *y*-axis) plotted against accuracies obtained with the original data (1×, *x*-axis) for each of seven tasks. Red, dGC; blue, iGC; dashed diagonal line, Line of equality (*x* = *y*). Right, N-way classification accuracies among all seven tasks with data sampled at 1×, 2×, and 3× of the original TR. Other conventions are the same as in ***F***. For ***B***, ***E***, and ***F***, accuracies correspond to highest values across all parcellations tested, and hyperparameter optimization was performed for ***B***. For ***C***, ***G***, and ***H***, accuracies correspond to Shirer et al. (2012) 14-network parcellation. For ***D***, accuracies correspond to Shirer et al. (2012) 90-node parcellation. Further details and control analyses are presented in Extended Data Figure 1-4, 1-5, 1-6, 1-7.

10.1523/ENEURO.0512-19.2019.f1-1 Figure 1-1 Task descriptions. Description of fMRI scans and tasks used in the analysis. Download Figure 1-1, DOC file.

We used five different brain parcellations based on anatomic atlas and four functional atlases (Extended Data Fig. 1-4). For the tasks versus resting-state classification based on GC connectivity (first section of Results), all five parcellations were used. Based on the classification performance in this analysis, we picked the three parcellations with the highest accuracies (90-node and 14-network parcellations of Shirer et al., 2012, and 96-network parcellation of Thomas Yeo et al., 2011), and these were used for the pairwise classification analysis of each task versus the other as well as the N-way task classification analyses. Analysis with averaging GC features across subjects (Fig. 1*D*) was performed with a 90-node parcellation (Shirer et al., 2012). Classification analyses with data purged of instantaneous correlations and unweighted digraph representations (second section of Results) were performed with the Shirer et al. (2012) 14-network parcellation. Analyses involving identifying task-generic and task-discriminative networks, as well as behavioral score predictions, based on GC features (last section of the Results) were performed with the Shirer et al. (2012) 14-network parcellation. Voxel time series were extracted using MATLAB and SPM 8 (Penny et al., 2007), and regional and network time series were computed by averaging the time series across all voxels in the respective region or network.

**Figure 2.**
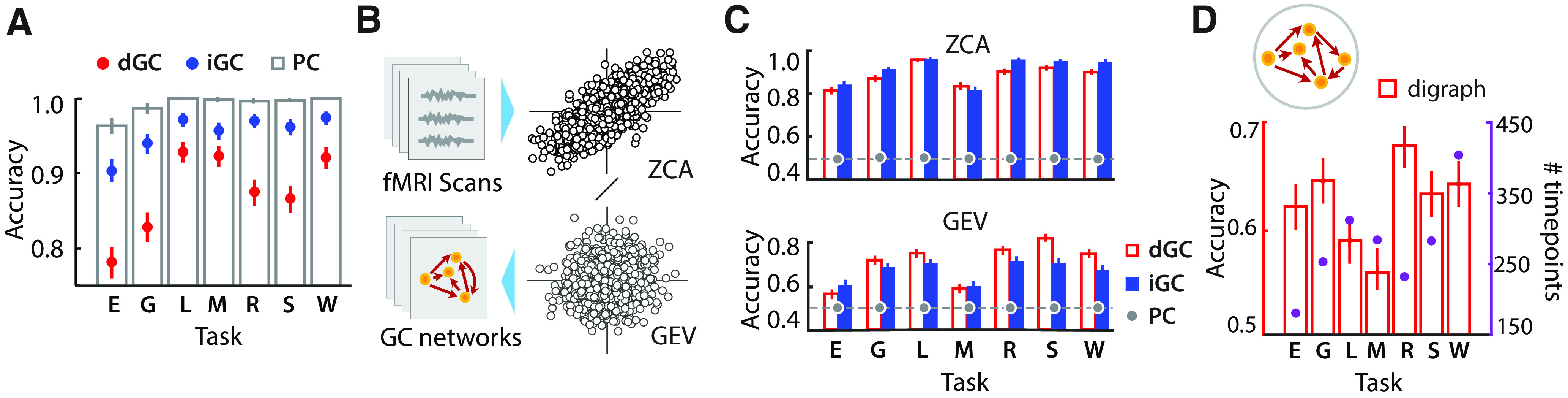
Classification accuracies with GC purged of instantaneous correlations. ***A***, Two-way task versus resting-state classification accuracies, based on PCs (gray unfilled bars). Numbers reported correspond to highest leave-one-out classification accuracies across parcellations, obtained with hyperparameter optimization. Corresponding accuracies for dGC (red dots) and iGC (blue dots) are shown for comparison. Other conventions are as in Figure 1*B*. ***B***, Schematic illustrating procedure for purging data of instantaneous correlations. fMRI regional time series were purged of instantaneous correlations by either whitening the data with ZCA, separately for each task and resting-state scan, or by projecting data into a space spanned by the GEVs, common to both task and resting-state scans. GC and PC were then estimated with the ZCA or GEV projections of the time series data, followed by classification analysis based on GC or PC connection strengths as features. ***C***, Top, Two-way task versus resting-state classification accuracies following ZCA-based decorrelation. Gray circles, Classification accuracies based on PC. Other conventions are as in Figure 1*B*; dashed line, chance accuracy (50%). Bottom, Same as in top panel, but for classification following GEV-based decorrelation. ***D***, Top, Schematic showing unweighted directed graph obtained from dGC; this digraph representation encodes only the dominant direction of connectivity, and not its magnitude. Bottom, Two-way task versus resting-state classification accuracies based on dGC digraph representations. Secondary ordinate (*y*-axis on the right), Number of scan timepoints for each task. ***C***, ***D***, GC features were estimated with the Shirer et al. (2012) 14-network parcellation.

10.1523/ENEURO.0512-19.2019.f1-2 Figure 1-2 Description of subtasks. Download Figure 1-2, DOC file.

**Figure 3.**
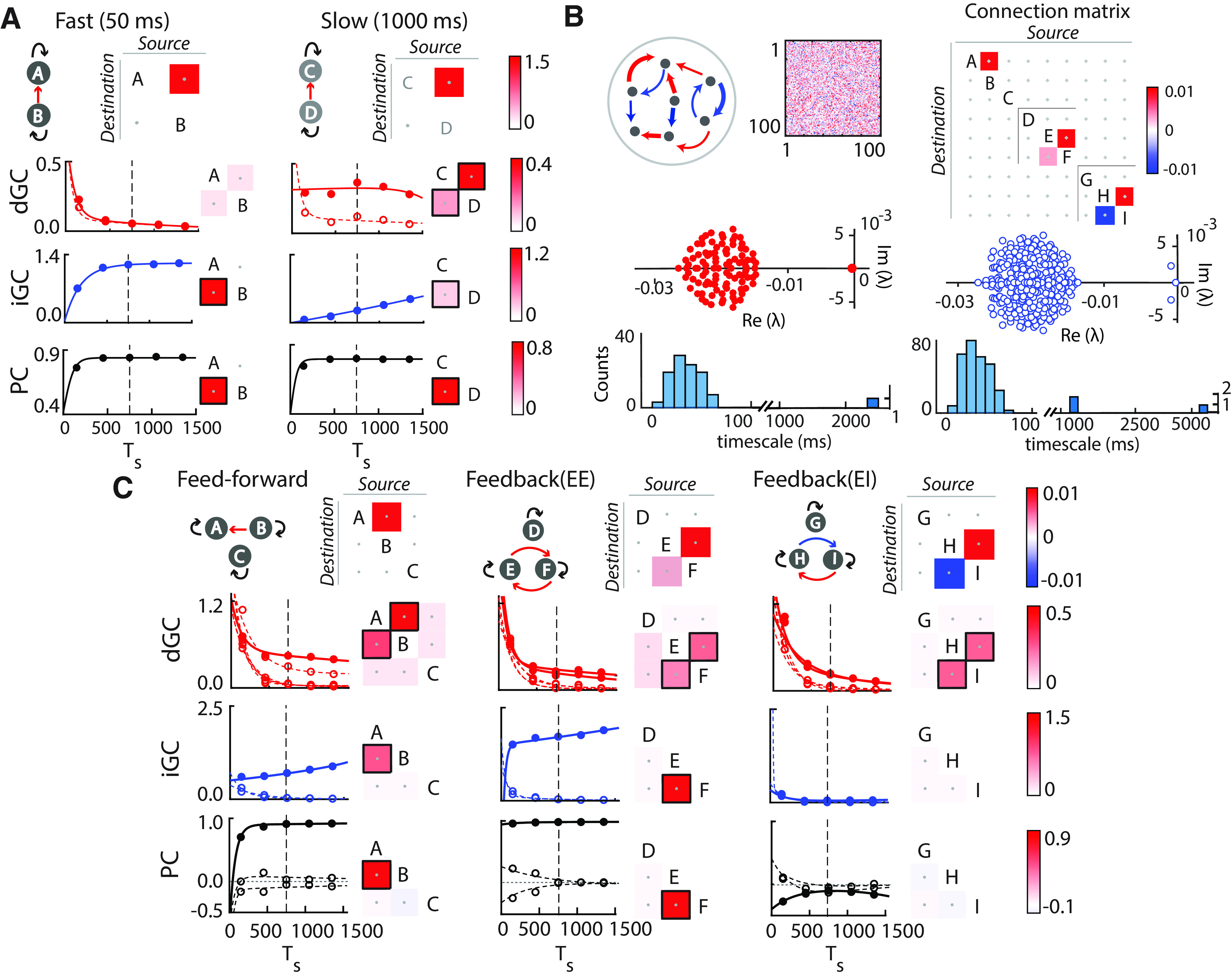
Robustness of GC estimates depends on network timescales in simulated hemodynamic data. ***A***, Top, Two-node networks with fast (50 ms; left) or slow (1000 ms; right) decay timescales of individual nodes (Extended Data Fig. 3-1*A*, parameters). Each subpanel shows ground truth connectivity either as a schematic (left) or a connectivity matrix (right). In the matrix, a nonzero entry at cell (i, j) corresponds to a connection from node j (source) to node i (destination). Bottom, dGC (red), iGC (blue), and PC (black) connection strengths as a function of sampling intervals. Filled circles and solid lines, Strengths of true connections and curve (biexponential) fits, respectively; open circles and dashed lines, strengths of spurious connections and curve fits, respectively; dashed vertical line, sampling interval of 750 ms, mimicking the TR of the fMRI data. Matrices to the right of each plot show GC connection strengths estimated at a sampling interval of 750 ms. Black squares surrounding matrix cells denote significant connections (Materials and Methods). For iGC and PC (symmetric connectivity), only the lower triangular matrix is shown, for clarity. ***B***, Top left, Schematic showing a cluster of neurons, each with a timescale of 50 ms, connected with sparse, random, net excitatory connectivity. Matrix, Connectivity among the 100 neurons in a representative cluster; red, excitatory connections; blue, inhibitory connections. Each such cluster forms one of the nine nodes in the simulated network. Top right, Connectivity among the nine nodes in the network (Extended Data Fig. 3-1*B*, parameters). Bottom left, Eigenspectrum (top) of a representative 100 neuron cluster, showing one slow emergent timescale corresponding to the real part of one eigenvalue close to zero. Histogram (bottom) showing timescales of all eigenmodes, with the slowest eigenmode at >2000 ms. Bottom right, Eigenspectrum (top) of subnetwork DEF exhibits multiple slow emergent timescales. Histogram (bottom) showing timescales of all eigenmodes, with three slow eigenmodes at ∼1000–6000 ms. ***C***, Same as in ***A***, but for simulated nine-node networks (Extended Data Fig. 3-1*B*, parameters). Left, Subnetwork ABC. Middle, Subnetwork DEF (see also Extended Data Fig. 3-2). Right, Subnetwork GHI. Other conventions are as in ***A***.

10.1523/ENEURO.0512-19.2019.f1-3 Figure 1-3 Subject identifiers. HCP IDs of 1000 subjects whose data were used in the analysis. Relational processing scans were not available for IDs marked in gray. Download Figure 1-3, DOC file.

**Figure 4.**
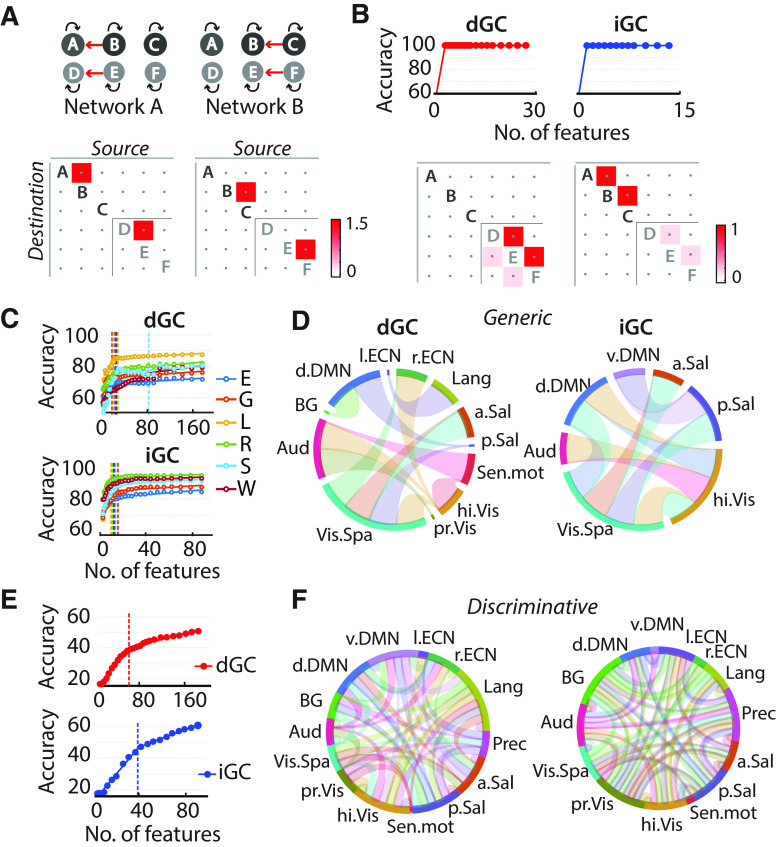
RFE identifies task-generic and task-discriminative networks based on GC connectivity. ***A***, Schematic showing two simulated networks each with fast (50 ms; ABC) and slow (1000 ms; DEF) subnetworks, with distinct connectivity patterns. Network activity was simulated for 375 s with a sampling interval of 5 ms, convolved with the hemodynamic response function and subsampled at 750 ms to yield 500 simulated timepoints. ***B***, Top, RFE curves, with classification accuracy as a function of the remaining features, for classification based on dGC (left) and iGC (right). Bottom, Maximally discriminative features following RFE based on dGC (left) and iGC (right). Entries denote average β weights across RFE iterations. ***C***, RFE curve for two-way classification of each of six tasks (all tasks except motor) versus rest, based on dGC (top) and iGC (bottom). Color conventions are as in Figure 1*D*. Data points, RFE accuracies; solid lines, piecewise linear fits; vertical dashed line, location of the elbow for each RFE curve. ***D***, Task–generic connections following task-versus-resting RFE, based on dGC (left) and iGC (right) features, using Shirer et al. (2012) 14-network parcellation (Extended Data Fig. 4-1, details); each network is indicated with a different color and a label. Directed dGC connections are shown as tapered links, broad at the source node and narrow at the destination node. Undirected iGC connections are shown as bidirectional links between the respective pair of nodes. Colors of the connections represent the color of the destination node. ***E***, Same as in ***C***, but for N-way classification across the six tasks. Color conventions are as in ***B***. ***F***, Same as in ***D***, but for task-discriminative connections (see also Extended Data Fig. 4-2), which maximally discriminated each task from the five others, following N-way RFE, based on dGC features (left) and iGC features (right). Other conventions are the same as in ***C***.

10.1523/ENEURO.0512-19.2019.f1-4 Figure 1-4 Parcellations used in the analysis. Download Figure 1-4, DOC file.

**Figure 5.**
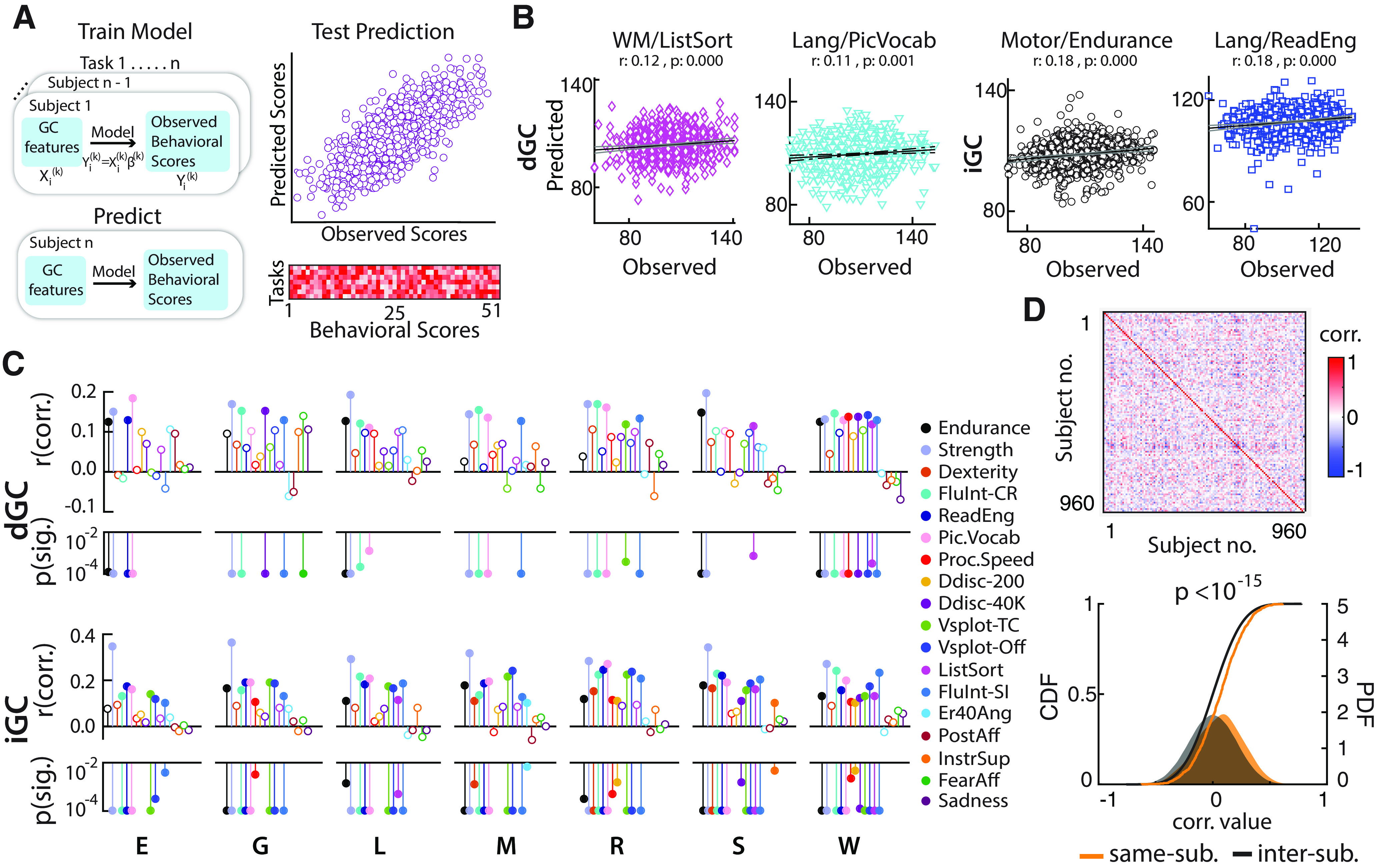
GC connectivity explains interindividual variations in behavioral scores. ***A***, Left, Schematic of behavioral score prediction analysis. GC connectivity strengths for each task were used as independent factors to predict behavioral scores using linear regression with a leave-one-out approach. Fifty-one different behavioral scores (Extended Data Fig. 5-1, descriptions) were predicted and compared against observed scores (top right), and their correlation values were plotted as a matrix (bottom right). ***B***, Exemplar score predictions based on dGC (left panels) and iGC (right panels). In order (from left to right): list sorting score predicted from working memory task dGC connectivity; picture vocabulary score from language task dGC connectivity; endurance score from motor task iGC connectivity; and reading score from language task iGC connectivity. ***C***, Top, Prediction statistics for selected scores based on dGC connectivity (all scores are shown in Extended Data Fig. 5-2). Correlation coefficients (*r* values) between the predicted and observed scores are plotted in the top half of each stem plot, and significance (*p* values) is plotted in the bottom half. Each score is denoted by a different color, and each subpanel shows predictions based on GC connectivity for a different task; stems with open symbols represent nonsignificant correlation coefficients, whose corresponding *p* values are not shown. *p* Values are floored at 10^−4^ for ease of visualization. Bottom, Same as in top panel, but predictions are based on iGC connectivity. ***D***, Top, Intersubject correlation matrix of composite behavioral scores. Row and column indices, subjects. Bottom, Cumulative distributions (solid lines) and density function estimates (filled area) of correlation coefficients between observed and predicted composite scores, for the same subject (yellow) or across different subjects (gray). Predictions were based on GC estimates from the relational and working memory tasks. *p* Value, Kolmogorov–Smirnov test.

10.1523/ENEURO.0512-19.2019.f1-5 Figure 1-5 GC classification accuracies for different parcellations and alternative classifiers. ***A***, Surface renderings showing the five anatomical and functional parcellations used in this study network (Extended Data Fig. 1-3). ***B***, Top row, Same as in Figure 1*B* (main text), but showing two-way task versus resting-state leave-one-out classification accuracies based on each of the five parcellations (***A***), each in one column. Second and third rows, Same as top row, but showing precision (second row) and recall (third row). Bottom row, Same as top row, but showing K-fold (10-fold) cross-validation accuracies. Other conventions are as in Figure 1*B* (main text). ***C***, Same as in Figure 1*B* (main text), but showing two-way task versus resting-state classification accuracies obtained using an SVM with an RBF (radial basis function) kernel (*y*-axis) against a conventional SVM (*x*-axis). Classification accuracies were computed with the Shirer et al. (2012) 14-network parcellation. Red and blue data: accuracies based on dGC and iGC features, respectively. Dashed diagonal line, Line of equality (*x* = *y*). ***D***, Top, Two-way classification accuracy for the working memory task versus resting-state classification, as a function of the number of scan timepoints used to estimate GC. Red dots, dGC; blue dots, iGC; curves, sigmoid fits; dashed horizontal line, chance accuracy (0.5). Bottom, Same as in the top panel, but two-way classification accuracies for distinguishing between two simulated networks (shown in Fig. 4*A*, main text). Other conventions are the same as in the left panel. Download Figure 1-5, EPS file.

10.1523/ENEURO.0512-19.2019.f1-6 Figure 1-6 Control analyses. ***A***, Comparison of average GC connection strengths of all subjects (even rows), and subjects who passed all tests of stationarity (odd rows), shown for each task (each column). Each 14 × 14 matrix depicts connections between all pairs of the 14 networks in the Shirer et al. (2012) parcellation. Entry in cell (i, j) corresponds to dGC connection from node j (source) to node i (destination) or iGC connections between nodes i and j. Source of connection at column, and destination at row. ***B***, Left, Comparison of two-way task versus resting-state classification accuracies, for the cohort of all subjects (*x*-axis) versus for subjects who passed all tests of stationarity (*y*-axis). Other conventions are the same as in Extended Data Figure 1-5C. Right, Comparison of N-way classification accuracies across all seven tasks, for all subjects (right bars) and for subjects who passed all tests of stationarity (left bars). Other conventions are the same as in Figure 1*F* (main text). ***C***, Left, Comparison of two-way task versus resting-state classification accuracies, without motion scrubbing (*x*-axis) versus after motion scrubbing (*y*-axis) for all subjects. Right, Comparison of N-way classification accuracies across all seven tasks, without motion scrubbing (right bars) and after motion scrubbing (left bars) for all subjects. Other conventions are the same as in ***B***. ***D***, Comparison of dGC connection strengths calculated using one-stage versus two-stage methods, for all seven tasks and resting state. Each point corresponds to the average strength, across subjects, of each one of the 182 dGC connections (***A***). Diagonal line, Line of equality (*x* = *y*). ***E***, Distribution, across all subjects, of correlation coefficients (*r* values) obtained by correlating one-stage versus two-stage dGC estimates across connections for each subject. Distributions for each of the seven tasks and resting state are shown in different colors. ***F***, Distributions, across all connections, of correlation coefficients (*r* values) obtained by correlating one-stage versus two-stage dGC estimates across subjects for each connection. Distributions for each of the seven tasks and resting state are shown in different colors. ***G***, Distributions of FD values (log-scale) across all tasks and resting scans of all 1000 subjects. Each color denotes one of the seven task (or resting) scans. Dotted line, Threshold FD value of 0.5 mm. For ***A–F***, accuracies and connectivity estimates were computed with the Shirer et al. (2012) 14-network parcellation. Download Figure 1-6, EPS file.

10.1523/ENEURO.0512-19.2019.f1-7 Figure 1-7 Number of subjects passing stationarity tests. Download Figure 1-7, DOC file.

We used parcellations with fewer, more coarse-grained regions, rather than fine-grained parcellations because Granger causality estimates were more reliable when the number of regions was fewer than the number of timepoints. Both task and resting scans were of sufficient duration (∼200–300 volumes) to permit robust GC estimation. Finally, we noticed that in some parcellations, there were overlapping voxels between some of the regions. To avoid mixing of signals, we assigned each overlapping voxel to the region to whose centroid it was closest, based on Euclidean distance.

### Estimating functional connectivity with GC

We modeled instantaneous and lag-based functional connectivity between brain regions using conditional Granger–Geweke causality (Geweke, 1984). The linear relationship between two multivariate signals, **x** and **y**, conditioned on a third multivariate signal, **z**, can be measured as the sum of linear feedback from **x** to **y** (Fx → y|z), linear feedback from **y** to **x** (Fy → x|z), and instantaneous linear feedback (Fx∘y|z; Geweke, 1984; Roebroeck et al., 2005). To quantify these linear relationships, we model the future of each time series in terms of their past values, using multivariate autoregressive (MVAR) modeling (Extended Data 1 Mathematical Note, Section S1, Eq. 1). MVAR model order was determined with the Akaike information criterion for each subject, and was typically 1. The MVAR model fit was used to estimate both an instantaneous connectivity matrix using iGC (Fx∘y|z) and a lag-based connectivity matrix using directed GC (dGC; Fx → y|z). Details are provided in Extended Data 1 Mathematical Note, Section S1. Because the minimum number of scans across datasets (176) exceeded the number of nodes in all parcellations used (90 nodes in the Shirer et al., 2012, parcellation), the GC estimation was well posed.

10.1523/ENEURO.0512-19.2019.ed1 Extended Data 1 Mathematical Note. The MATLAB codes to reproduce the results are available at https://figshare.com/s/9d9131a6780fc8197cf1. Separate folders correspond to each figure, and subfolders contain scripts for generating each panel in the respective figure. The filenames are alphabetically ordered to provide a sequence for running the scripts. The Multivariate Granger Causality toolbox (mvgc_v1.0; available at http://users.sussex.ac.uk/~lionelb/downloads/mvgc_v1.0.zip) is a prerequisite. Data necessary to run the scripts (both input and output) are placed in a “data” subfolder within each figure folder. Download Extended Data 1, PDF file.

Briefly, Fx → y|z is a measure of the improvement in the ability to predict the future values of **y** given the past values of **x**, over and above what can be predicted from the past values of **z** and **y**, itself (and vice versa for Fy → x|z). Fx∘y|z, on the other hand, measures the instantaneous influence between **x** and **y** conditioned on **z** (Extended Data 1 Mathematical Note, Section S1). We refer to Fx∘y|z, as iGC, and Fx → y|z and Fy → x|z as lag-based GC or dGC, with the direction of the influence (**x** to **y** or vice versa) being indicated by the arrow. The “full” measure of linear dependence and feedback Fx,y|z is given by the following: Fx,y|z = Fx → y|z + Fy → x|z + Fx∘y|z. Fx,y|z measures the complete conditional linear dependence between two time series. If, at a given instant, no aspect of one time series can be explained by a linear model containing all the values (past and present) of the other, Fx,y|z will evaluate to zero (Roebroeck et al., 2005).

### Classification with linear Support Vector Machines based on GC connectivity

The connection strengths of the estimated GC functional connectivity matrices were used as feature vectors with a linear classifier based on Support Vector Machines (SVMs) for high-dimensional predictor data. For a parcellation with *n* ROIs, the number of features for iGC-based classification was n(n - 1)/2 (upper triangular portion of the symmetric *n* × *n* iGC matrix) and for dGC-based classification it was *n*^2^ − *n* (all entries of the *n* × *n* dGC matrix, excluding self-connections on the main diagonal). Based on these functional connectivity features, we asked whether we could reliably distinguish each task condition from resting state (e.g., language vs resting) or each task condition from the other.

For pairwise classification of resting state scans versus each task, we used the MATLAB fitclinear function, optimizing hyperparameters using a fivefold approach, as follows: by estimating hyperparameters with five sets of 200 subjects in turn, and by measuring classification accuracies with the remaining 800 subjects. Classification performance was assessed with leave-one-out and 10-fold cross-validation. We also assessed the significance of the classification accuracy with permutation testing (see Materials and Methods). In simulations, we observed that the magnitude of GC estimates varied based on the number of timepoints used in the estimation. To prevent this difference in the number of timepoints from biasing classification performance, each scan was truncated to a common minimum number of time samples across the respective scans being classified (task, resting) before estimating GC. For each subject, GC connectivity was estimated independently for the two scan runs (left-to-right and right-to-left phase-encoding runs) and was averaged across the runs. The hyperparameters optimized included the regularization parameter, regularization method (ridge/lasso), and the learner (linear regression model, svm/logistic) using the OptimizeHyperparameters option to the fitclinear function. Hyperparameter optimization was performed only for task versus rest classifications, but not for subject feature averaging, task versus task, or N-way classification analyses.

For pairwise classification of each vs the other, default hyperparameters were used in the fitclinear function and classification performance was assessed with leave-one-out cross-validation. For N-way classification, we used the MATLAB fitcecoc function, which is based on error-correcting output codes, and fits multiclass models for SVMs. Briefly, the function implemented a one-versus-all coding design, for which seven (number of classes in multiclass classification) binary learners were trained. For each binary learner, one class was assigned a positive label and the rest were assigned negative labels. This design exhausts all combinations of positive class assignments. Classification performance in N-way classification was assessed with leave-one-out cross-validation. For each classification analysis mentioned above, task scans were truncated to the common minimum number of time samples across each set of scans, before estimating GC.

### Classification based on GC connectivity across subtasks and with subsampled data

Tasks in the HCP data were run as a block design, alternating between various conditions (subtasks). We tested whether GC connectivity would be able to classify among subtasks within each task (Extended Data Fig. 1-2). fMRI time series corresponding to each subtask was obtained by concatenating blocks of fMRI task time series pertaining to the respective subtask; the temporal order across blocks was preserved while concatenating the data. We also ensured that data at the conjunction of two successive blocks, which represented noncontiguous timepoints, were not used for GC estimation. The two subtasks to be classified were then truncated to have the same number of timepoints. GC estimation and pairwise classification across subtasks were performed with the procedure described in the previous section. The Shirer et al. (2012) 14-network parcellation was used for these analyses. For the motor task, time series for the left and right finger movement blocks were combined into a “hand” movement subtask, and left and right toe movement blocks were combined into a “foot” movement subtask.

We also tested whether GC on fMRI data sampled at slower rates would suffice to classify among task and resting states. We obtained time series downsampled at 2× the original sampling interval by removing data at even-numbered sample points and retaining data at odd-numbered sample points (k = 1, 3, 5…). The even-sample point data were appended the end of odd-sample data series, thereby retaining the overall number of data points in the original time series. Again, we ensured that data at the conjunction of the odd- and even-sampled data series (last odd-sampled point and first even-sampled point), which represented noncontiguous data points, were not used for GC estimation. Similarly, we obtained time series downsampled at 3× the original sampling interval by removing every third data point, starting with the second or third data point, and concatenating these time series to retain the overall number of data points in the original time series. As before, GC estimation and pairwise classification was performed with the procedure described in the previous section

### Permutation testing of classifier accuracies

We performed permutation tests for evaluating the statistical significance of classifier performance, using the method outlined in the study by Ojala and Garriga (2010). The test involved permuting task labels independently for each subject and computing a null distribution of 10-fold cross-validation accuracy. We used 1000 surrogates and assessed the significance of each empirically estimated 10-fold cross-validation accuracy values for dGC and iGC, based on the proportion of samples in the null distribution, which were greater than the cross-validation accuracy estimated from the data. We conducted these analyses for the tasks versus resting-state classifications, N-way task classification, classification analyses after purging instantaneous correlations, and classifications based on digraph features, separately for the two metrics (dGC and iGC).

### Testing for data stationarity and goodness of MVAR model fit

Computing GC based on VAR modeling assumes that the time series represent a stationary process. Four different tests were performed to test whether the MVAR model provided a valid and adequate fit to the data (Extended Data Fig. 1-7). We performed these tests for parcellated time series using scripts provided in the multivariate Granger causality (MVGC) toolbox (Barnett and Seth, 2014). First, we checked for the stability of the MVAR model fit by computing the logarithm of the spectral radius using the *var_specrad()* function. A negative value was taken to indicate a stable fit. Second, we assessed the consistency of the model fit, which quantifies what proportion of the correlation structure in data are accounted for by the VAR model, using the *consistency()* function. We adopted a threshold of 80% (or above) for both task and resting time series to consider the data to have passed the test for consistency (Barnett and Seth, 2014). Third, we evaluated the whiteness of residuals based on the Durbin–Watson test for the absence of serial correlation of VAR residuals, using the *whiteness()* function. Values of the Durbin–Watson statistic <1 or >3 signify a strong positive or negative correlation, respectively, among the residuals (Barnett and Seth, 2014). Subjects for whom the Durbin–Watson statistic lay between 1 and 3 for >90% of the regional time series, for both task and resting-state data, were considered to have passed the test. Fourth, we checked for stationarity based on the augmented Dicky–Fuller unit-root test (ADF), using the *mvgc_adf()* function. As in the previous case, subjects for whom the ADF test statistic was less than its critical value for >90% of the regional time series, for both task and resting-state data, were considered to have passed the test.

### Control for motion artifacts

We checked whether systematic differences in motion artifacts could contribute to the superlative classification accuracies observed with GC. For this, we calculated framewise displacement (FD; Power et al., 2012) as the sum of temporal derivatives of translational and rotational displacement along the three (*x*, *y*, and *z*) axes in millimeters, with the estimated motion parameters provided by HCP. Frames with FD > 0.5 mm were considered “misaligned” and were discarded (“scrubbed”) while estimating GC values. Because dGC is estimated based on lagged correlations, we also discarded one frame before and after every misaligned frame (the AR model order was typically 1 for these data). We then repeated the SVM-based two-way classification of resting state from the seven different task states, with GC features estimated on the “motion scrubbed” data; we also repeated N-way classification among the seven tasks. Comparison of classification (cross-validated) accuracies with and without motion scrubbing, across all 1000 subjects, is shown in Extended Data Figure 1-6*C*.

### Classification based on BOLD series

We tested how well the BOLD signal itself would classify among tasks, based on the mean and SD of fMRI time series of each region, based on the Shirer et al. (2012) parcellation. Regional time series were truncated to a common minimum number of timepoints for a pair of task and resting-state scans. LR (left-to-right) and RL (right-to-left) phase-encoded data time series were concatenated, and the mean and SD were computed, for each of the 14 ROIs, providing 28 features for classification. Similarly, for N-way classification, time series of all tasks were truncated to the common minimum available number of timepoints across tasks, before computing the mean and SD. Based on these 28 features, we sought to classify, as before, the resting state from each task (two-way classification) and also among tasks (N-way classification).

### Functional connectivity estimation and classification with partial correlations

We compared the performance of classification based on GC measures with that based on PCs. Partial correlations were computed based on the inverse of the covariance matrix, as outlined previously (Marrelec et al., 2006; Ryali et al., 2012). Like iGC, the PC connectivity matrix is undirected and symmetric. Therefore, only the upper triangular portion of the matrix, including (*n* * (*n* − 1)/2) PC weights, was used as features in the classification analyses. Classification and cross-validation analyses followed the procedures described in the Materials and Methods section Classification with linear support vector machines based on GC connectivity.

PC connectivity performed consistently better than GC connectivity for classifying task from resting state (Fig. 2*A*). We propose the following analytical explanation for this observation: PC, an estimator based on instantaneous covariance, is less susceptible to noise than GC, which is based on lagged covariance. This is due to the fact that the estimation of lagged covariance is susceptible to errors from noise at multiple timepoints. For illustration, consider a time series generated by a VAR(1) model as follows: x(t) = A x(t - 1) + e(t). The lagged (lag-1) covariance matrix (Σ_1_) is estimated from the data as follows:

$$\text{E}\;[x(\text{t})\;x{\left(\text{t}\;\text{-}\;\text{1}\right)}^{\text{T}}]\;\text{=}\;\text{E}\;[(Ax(\text{t}\;\text{-}\;\text{1})\;\text{+}\;e(\text{t}))\;x{\left(\text{t}\;\text{-}\;\text{1}\right)}^{\text{T}}]=A\;\text{E}[x(\text{t}\;\text{-}\;\text{1})\;x{\left(\text{t}\;\text{-}\;\text{1}\right)}^{\text{T}}]+\text{E}\;[e(\text{t})\;x{\left(\text{t}\;\text{-}\;\text{1}\right)}^{\text{T}}]$$

Thus, when estimating the lagged covariance, the variance of the interaction term $\text{E}\;[e(\text{t})\;x{\left(\text{t}\;\text{-}\;\text{1}\right)}^{\text{T}}]$ (second term on the right-hand side) contributes to the variance of Σ_1_ in addition to the variance in computing the instantaneous covariance $\text{E}[x(\text{t}\;\text{-}\;\text{1})\;x{\left(\text{t}\;\text{-}\;\text{1}\right)}^{\text{T}}]$ (first term on the right-hand side).

### Classification based on GC connectivity in zero-lag correlation purged data

To test the complementarity of information conveyed by GC functional versus functional connectivity based on instantaneous correlations, we decorrelated the regional time series data to purge them of instantaneous correlations. We adopted the following two approaches for this purpose: (1) zero-phase component analysis (ZCA) and (2) generalized eigenvalue (GEV) decomposition.

#### ZCA

Consider the demeaned *t *×* r* data matrix **X** of regional time series with *t* timepoints and *r* regions, with covariance matrix **C**. Decorrelating the data, to remove correlations among the columns of **X**, is achieved with a whitening transformation. A common whitening transformation is based on principal component analysis (PCA), as follows: **Y = W_PCA_X**, with **W_PCA_ = D^−1/2^E^⊤^**, where **D** is a diagonal matrix, with the eigenvalues of **C** on its diagonals, and the columns of **E** contain the eigenvectors of **C**. While the PCA transformation effectively decorrelates regional time series, there is no way to ensure one-to-one correspondence of the whitened dimensions across subjects, rendering subsequent classification analysis challenging. Consequently, here we chose a different whitening transformation based on ZCA, also known as the Mahalanobis transformation. Based on this transformation, whitening is achieved as: **Y = W_ZCA_X,** with **W_ZCA_ = ED^−1/2^ E^⊤^ = C^−1/2^**. A particular advantage of the ZCA transformation is that it yields whitened data that are as close as possible to the original data, in a least-squares sense (Kessy et al., 2018). Therefore, each subject’s data are projected on to a set of dimensions that are most closely aligned with the underlying regional time series dimensions. Because the regions exhibit spatial correspondence across subjects (due to fMRI spatial normalization), the ZCA dimensions possess a natural, one-to-one correspondence across subjects, permitting subsequent classification. Before classification analysis, ZCA dimensions were identified for each subject, separately for task and resting datasets. Regional time series for task and resting data were independently decorrelated by projecting onto their respective ZCA dimensions. GC (and PC) functional connectivity was estimated based on these decorrelated time series, followed by classification analysis, as described previously (Classification with linear support vector machines based on GC connectivity, in Materials and Methods). As proof that the ZCA transformation was working effectively, classification accuracy based on PC (an instantaneous correlation measure) computed from ZCA components was at chance across all tasks (Fig. 2*C*, top).

#### GEV

Although ZCA effectively purged correlations from the data, for the subsequent classification analyses task and resting-state data were projected onto different, respective ZCA dimensions. Thus, the above-chance versus resting-state classification accuracy with GC features derived from ZCA components (Fig. 2*C*, top) could perhaps be explained by, for example, systematic differences with how reliably ZCA dimensions were estimated across task and resting-state scans. We therefore sought an approach that could project both task and resting data into the same dimension while simultaneously decorrelating both. Such joint decorrelation may be achieved by projecting the data on to the generalized eigenvectors of the covariance matrices of the two datasets (Karampatziakis and Mineiro, 2014). Let **C_T_** and **C_R_** denote the covariance matrices of the task and resting datasets, respectively. The generalized eigenvectors of these two symmetric matrices are given by the columns of **G** = **E_T_ D_T_^−1/2^ E_R_**, where, as before, **D_T_** is a diagonal matrix, with the eigenvalues of **C_T_**on its diagonals, and the columns of **E_R_**and **E_T_**contain the eigenvectors of **C_R_**and **C_T_**, respectively. It can be readily verified that **G** ^T^ **C_T_G** and **G** ^T^ **C_R_G** are both diagonal matrices. Therefore, **G** is a matrix that jointly diagonalizes both **C_T_** and **C_R_** and projecting either task or resting-state data into the columns of **G** decorrelates the respective time series. So, for these analyses, the regional time series for the task and resting-state conditions for each subject were jointly decorrelated by projecting them onto a single space spanned by the generalized eigenvectors. This was followed by classification analysis with GC features obtained from the decorrelated time series. As before, we confirmed the effectiveness of the decorrelation by computing classification accuracy based on PC from GEV components, which was at chance across all tasks (Fig. 2*C*, bottom).

### Classification based on unweighted digraph representations of GC connectivity

An unweighted directed graph (digraph) network representation shows the dominant direction (but not magnitude) of functional connectivity among brain regions. Obtaining significant directed connections with dGC is challenging due to the number of multiple comparisons required for testing *n*^2^ − n connections. To identify significant directed connections, overcoming the multiple comparisons problem, we first subtracted the dGC connectivity matrix from its transpose and then applied the following two-stage procedure. In the first stage, the 1000 subjects were divided into five folds. For each two-way task versus resting-state classification, recursive feature elimination (RFE; described in GC feature selection based on recursive feature elimination) was performed based on dGC features of subjects from one fold (i.e., with 200 subjects). A minimal set of connection features identified by RFE and their corresponding symmetric counterparts were then used in the subsequent analyses; we term these connections K; the cardinality of K (the number of significant connections) was typically in the range of 2–86 (2.5th to 97.5th percentile). In the second stage, we identified statistically significant connections among these K features alone. For each of the subjects in the four remaining folds (i.e., 800 subjects), a null distribution for the dGC values of the features in K was obtained by estimating dGC following phase scrambling of the time series (Ryali et al., 2011). Next, we identified significant connections based on dGC values that occurred at the tail of the null distribution; the threshold for significant connections was determined based on a *p* value of 0.05 with a Bonferroni correction for multiple comparisons. Classification performance based on digraph features was assessed with leave-one-out cross-validation.

### GC connectivity in simulated fMRI time series

To test the ability of GC measures to reliably recover functional interactions at different timescales, we simulated fMRI time series for model networks. Simulated fMRI time series were generated using a two-stage model. The first stage involved modeling latent neural dynamics with a stochastic, linear vector differential equation given by the following:

τdr/dt = -r + Wr + ε,

where **r** is the multivariate neural state variable representing the state of each neuron (or node) in the network (an *N* × 1 vector, with *N* being the number of neurons), d**r**/dt is its temporal derivative, *W* is the neural (“ground truth”) connectivity matrix (dimension *N* × *N*), τ is the time constant of each neuron (or node), and **ε** is independent and identically distributed Gaussian noise (*N*(0, Σ)), with Σ = *I*_N_ (*N* × *N* identity matrix). Although this model does not explicitly incorporate signal propagation delays, such vector Ornstein–Uhlenbeck models rank, arguably, among the most common models used for simulating neural and fMRI time series, in many previous studies (Smith et al., 2011; Seth et al., 2013; Barnett and Seth, 2017).The multivariate time series **r**(*t*), sampled at discrete timepoints *r*(*k*Δ) with a sampling rate of Δ, were generated based on the discrete time (1-lag) connectivity matrix A(Δ) and a residual noise intensity Σ(Δ), as shown here:

$$A(\Delta )\;\text{=}\;e^{\Delta A};\;\Sigma (\Delta )\;\text{=}\;(\text{1/}\Delta )\;(\Gamma (\text{0})\;\text{-}\;e^{\Delta A}\Gamma (\text{0})\;e^{\Delta A\prime }),$$

where $A\;\text{=}\;(\text{1/}\tau )\;(W\;\text{-}\;I_{\text{N}})$, *e^A^* denotes the matrix exponential, *A′* is the transpose of *A*, and Γ(0) is the zero lag autocovariance that satisfies the continuous time Lyapunov equation AΓ(0) + Γ(0)A′ + Σ = 0 (Seth et al., 2013). In the second stage, the latent neural dynamics were convolved with the hemodynamic response function (HRF) to obtain the simulated fMRI time series: **y **=** ***H* ⊗**x**, where *H* is the canonical hemodynamic response function (hrf; simulated with *spm_hrf* in SPM8), ⊗ is the convolution operation, and **y** is the simulated fMRI time series. Finally, following convolution with the hrf, the data were downsampled to 750 ms to mimic the repeat time (TR) of the HCP fMRI scans used in this study. The same model was used for the different simulations used in the manuscript (third section of the Results). The parameters for the two-node simulations and for the nine-node (100 neurons per node) simulations are described in Extended Data Figure 3-1.

10.1523/ENEURO.0512-19.2019.f3-1 Figure 3-1 Parameters of simulated networks. Parameters of two-node and nine-node networks. Download Figure 3-1, DOC file.

For the two-node simulations (Fig. 3*A*), iGC and dGC values were estimated by simulating the network for 200 timepoints, averaged across 25 repetitions. The nine-node simulations (Fig. 3*B*,*C*) were performed with a 900-neuron network, with 100 neurons per node. Each node had sparse, random excitatory/inhibitory connectivity among its neurons (Extended Data Fig. 3-1, parameters), whereas only 5% of neurons in each node were involved in internode connections, to mimic sparse, long-range connectivity in the neocortex (Knösche and Tittgemeyer, 2011). The network was simulated for 200 timepoints, and time series from all (100) neurons in each node were averaged to generate nine-node time series. iGC and dGC values were estimated from the node time series and averaged across 10 independent repetitions. Significance was assessed with a bootstrap approach that involved generating 1000 surrogates by phase scrambling the node time series to yield a null distribution of GC values (Ryali et al., 2011), followed by a Benjamini–Hochberg correction for multiple comparisons.

10.1523/ENEURO.0512-19.2019.f3-2 Figure 3-2 Relationship among network connectivity, GC, and partial correlations. ***A***, Same as in Figure 3*C* (middle column panel; main text) except for a network with balanced E-E feedback (middle). Matrix shows the connections estimated at a sampling interval of 750 ms (bottom). Other conventions are the same as in Figure 3*C* (main text). ***B***, Left, Schematic of a two-node network simulated with a discrete time vector autoregressive model. c and d denote the strength of internode connections, and a and b denote the strength of recurrent connections within each node. Here, for simplicity, we assume a = b. Right, Top, Variation of zero-lag covariance (σ_12_), which is the basis of computing PC, with varying values of c + d for three different values of a. Note that σ_12_ is zero when c = −d, regardless of a. Right, Bottom, Example simulations of node dynamics for c = −0.2 and d = 0.2. ***C***, Covariation of iGC (blue triangles), PC (filled black circles), and PC covariance (K; open squares) with iGC covariance (Y) for simulations with a first-order vector autoregressive (AR) model, with both instantaneous and lag-based connectivity (Extended Data Mathematical Note, Section S3, Eqs. 11, 23). Open circles, PC covariance (K) for a system with no lag-based connectivity (AR coefficients zero). ***D***, Top, Difference in dGC estimates (ΔdGC) between connection in actual direction and connection in the reverse direction, plotted against different SDs in onset latencies (σL). Each color denotes one particular scenario of differences in onset HRF latencies (see text for details). Columns 1 and 2, Network A; columns 3 and 4, network B; odd columns, fast timescale (50 ms) subnetwork (ABC); even columns, slow timescale (1000 ms) subnetwork (DEF; refer to Fig. 4*A*, main text). Bottom, Same as in top panel, but for iGC estimates. Error bars indicate SEM. ***E***, Leftmost column, Ground truth connectivity matrix for the two networks. Other columns, Same as in Figure 4*B* (main text), but showing RFE curves (top subpanel) and maximally discriminative features following RFE (bottom subpanel). Rows 1 and 2, RFE based on dGC features; rows 3 and 4, RFE based on iGC features; filled circles, number of features at the elbow of each RFE curve. Each column corresponds to one of the four scenarios of onset latency differences (see ***D*** and text for details). All panels, HRF onsets sampled from a distribution with σL = 0.4 s. Other conventions are the same as in Figure 4*B* (main text). Download Figure 3-2, EPS file.

Simulations comparing PC and iGC connectivity (Extended Data Fig. 3-2*B*,*C*) were performed as follows: we simulated a seven-node network with a 1-lag VAR model of the form **X**_k_ = *A* **X**_k−1_ + **ɛ**_k_, where **X**_k_ is the state of the discrete time process at discrete timestep k, *A* is the connectivity matrix, and **ɛ** is Gaussian noise with covariance matrix Σ_d_. *A* was chosen to be a random matrix with spectral radius <1 to ensure stability. Σ was chosen such that the covariance between every pair of residuals was zero (independent residuals) except for the first two residuals. The correlation between these residuals, ɛ^1^ and ɛ^2^, was parametrically varied between −1.0 and 1.0 to systematically vary the strength of iGC connectivity. Note that, under this model, iGC between X^1^ and X^2^ vanishes only if ɛ^1^ and ɛ^2^ are uncorrelated (Geweke, 1984).

### GC feature selection based on RFE

We performed features selection for analyses reported in Figures 2*D*, and 4, *B* and *C*, Extended Data Figure 4-2*B*, and Extended Data Figure 1-4*E*, based on RFE. RFE identifies a minimal set of features, which provide maximal cross-validation accuracy (Guyon and Elisseeff, 2003). Here, we implemented a two-level algorithm, described previously (De Martino et al., 2008). First, the data were divided into *N*_1_ (here, 10) folds. Of these, *N*_1_ − 1 folds were used as “training” data, and one fold was reserved as “test” data for quantifying the generalization performance of the classifier. Training data were pooled and further divided into *N*_2_ (here, 5) folds. The SVM classifier was then trained on *N*_2_ − 1 folds (leaving out one fold), and discriminative weights were obtained. The above procedure was repeated *N*_2_ times by leaving out each fold, in turn. Average weights were then computed by averaging the absolute values of the discriminative weights across the *N*_2_ runs. Next, 10% of the features (connections) contributing the lowest average weights were discarded, and the classifier was trained again with only the retained set of features. This procedure of feature selection and training was repeated until no more features remained. At this stage, the generalization performance for every set of retained features (each “RFE level”) was assessed using the left out test data. The entire procedure was repeated *N*_1_ times by leaving out each fold of the original data, in turn, as test data. Final generalization performances and discriminative weights of each RFE level were obtained as the average over *N*_1_ folds. We selected the set of connections at the RFE level at which the generalization performance reached an “elbow”: a minimal set of connections at which generalization performance dipped dramatically below its maximal level. To identify this elbow (e), we used a custom elbow-fitting procedure, requiring a piecewise linear fit to the RFE curve, based on two lines, one for “*x* > e” and another for “*x* ≤ e,” with the first line required to have a higher slope than the second. The first point in each RFE curve was excluded from the higher slope line fit (Fig. 4*C*,*E*, Extended Data Fig. 4-2*B*). RFE was typically repeated five times before determining peak accuracy and corresponding features.

10.1523/ENEURO.0512-19.2019.f4-1 Figure 4-1 Network labels in the Shirer et al. (2012) 14-network parcellation. Download Figure 4-1, DOC file.

10.1523/ENEURO.0512-19.2019.f4-2 Figure 4-2 Task-generic and task-discriminative connections based on PCs. ***A***, Task-discriminative connections based on dGC (top row), iGC (middle row), and PC (last row). Other conventions are as in Extended Data Figure 1-6A. ***B***, Top, Same as in Figure 4*C* (main text), but for RFE based on PC. Bottom, Same as in Figure 4*E* (main text), but for RFE based on PC. ***C***, Same as in Figure 4*D* (main text), but for task-generic connections based on PC. ***D***, Same as in Figure 4*F* (main text), but for task-discriminative connections based on PC. Download Figure 4-2, EPS file.

### Simulating hemodynamic lag variations across nodes

We simulated systematic differences in hemodynamic lags across nodes by varying the onset parameter of the *spm_hrf* function (SPM8; Penny et al., 2007). For network configurations A and B described in Figure 4*A*, we simulated the following four scenarios: (1) same mean HRF onset (μ_L_ = 3 s) across nodes; (2) source node HRF onset lagging the destination node by 1 s ($\mu_{\text{L}-\mathrm{src}}\;>\;\mu_{\text{L-dst}}$); (3) source node HRF onset leading destination node by 1 s ($\mu_{\text{L}-\mathrm{src}}\;>\;\mu_{\text{L-dst}}$); and (4) mixed latencies of lead and lag across source and destination nodes (see next section). GC was estimated for 100 simulated participants by sampling onset latencies for each of the six nodes (A–F) from normal distributions (truncated to have only positive latency values), over a range of different SDs (σ_L_ = 0–1 s, in steps of 0.2 s). Onset latencies were sampled independently across participants, but were sampled such that the relative latency between each pair of source and destination nodes, across corresponding network configurations, remained the same for each participant. For example, if the onset latency difference between nodes A and B was 0.7 s ($\mu_{\text{L-B}}\;\text{-}\;\mu_{\text{L-A}}\;\text{=}\;\text{0}.\text{7}\;\text{s}$) for a particular subject, the same difference in onset latency was also maintained between nodes B and C ($\mu_{\text{L-C}}\;\text{-}\;\mu_{L\text{-B}}\;\text{=}\;\text{0}.\text{7}\;\text{s}$). For simulations with mixed latencies (case 4), 50% of simulated participants had onset latencies drawn from distributions with the source node lagging the destination node (case 2) and the remaining 50% with the source node leading the destination node (case 3). GC values were averaged over five runs for each simulated participant. Finally, we performed RFE to identify key connections that distinguished the two network configurations (same procedure as in Fig. 4*B*). Connections weights of the most discriminative connections following RFE are shown in Extended Data Figure 3-2*E* (for $\sigma_{\text{L}}\;\text{=}\;\text{0}.\text{4}\;\text{s}$). Difference of dGC connection strengths, as well as iGC connection strengths, for various values of σ_L_, are shown in Extended Data Figure 3-2*D*.

### Identifying task-generic and task-discriminative GC connections

To identify a minimal set of connections that occurred consistently across tasks (“task-generic” connections), we adopted the following approach. We performed RFE analysis for task versus resting-state classification for each of the six tasks (all tasks except motor); we expected each of these tasks to recruit common cognitive control mechanisms. We then performed a binomial test to identify connections that were consistently activated across tasks. Briefly, the presence or absence of a connection in the set of RFE features for a given task versus resting-state classification was considered as a Bernoulli trial, with probability of success (its presence), *p* being the mean number of RFE features identified across all six classifications. The number of trials *n* was the number of task versus resting-state classifications (here *n* = 6). The probability of a randomly picked connection being present in more than *k* such RFE sets is given by the cumulative distribution function for the binomial distribution *F(k; n, p).* Significant connections were identified as those that occurred in *k* or more tasks, with threshold at the *p* = 0.05 level.

To identify a minimal set of connections that maximally differed across tasks (“task-discriminative” connections), we used RFE with an N-way classifier to classify among all six tasks (again, except the motor task). The N-way classifier is based on training *n* (here, 6) one-versus-all binary learners. At the second level of the RFE procedure described above, average weights were computed for each of these *n* binary learners by averaging the absolute values of the discriminative weights across the *N*_2_ runs. Next, a set of features obtained by taking a union of 1% of the features (connections) contributing the lowest average weights in each learner was discarded, and the classifier was trained again with only the retained set of features.

While quantifying the overlap between task-generic and task-discriminating connections identified separately for dGC, iGC and PC, we converted the dGC matrix to a lower triangular matrix by reflecting all connections about the main diagonal. The degree of overlap between PC and GC connections was quantified as the number of overlapping connections as proportion of the total number of connections identified by PC. We then computed a null distribution of the degree of overlap by randomly permuting the connection identities within each matrix, while preserving the overall number of connections in each matrix, and generating 1000 surrogate samples. The significance of the overlap of task-generic or task-discriminating connections between each pair of metrics (PC-dGC or PC-iGC) was quantified as the fraction of overlapping connections in the data that exceeded this null distribution.

### Predicting behavioral scores based on GC connectivity

We asked whether interindividual differences in GC connectivity would be relevant for predicting interindividual differences in behavioral scores. HCP provides a well validated battery of behavioral scores assessed with a wide range of cognitive tasks. The task battery is based on the NIH Toolbox for Assessment of Neurologic and Behavioral function (Gershon et al., 2013), which was developed to create a uniform set of measures for rapid data collection in large cohorts. The toolbox includes assessments of cognitive, emotional, motor, and sensory processing scores in healthy individuals. We preselected, based on domain knowledge, a specific subset of 51 scores for these analyses, using age-adjusted scores, wherever available (Extended Data Fig. 5-1). Next, we sought to predict subjects’ behavioral scores based on GC connectivity with an established leave-one-out approach (Tavor et al., 2016). Briefly, we used linear regression to predict behavioral scores using, as features, GC estimates of functional connectivity, separately for iGC (91 features or connections) and dGC (182 features). The leave-one-out analysis was performed such that the support vector regressor was fit on all but one subject, and the learned β weights were used to obtain predictions of the left-out subject’s behavioral score, using that subject’s own GC connectivity weights. Predicted scores were correlated with the actual scores using robust correlations (“percentage-bend” correlations; Wilcox, 1994).

10.1523/ENEURO.0512-19.2019.f5-1 Figure 5-1 Behavioral scores and descriptions. Download Figure 5-1, DOC file.

Next, we asked whether GC connectivity could identify an individual based on a composite marker of her/his behavioral scores. Because 40 subjects did not have a full complement of behavioral scores, data from the remaining 960 subjects were included in this analysis. The 51 behavioral scores were each *z*-scored across subjects and formatted into a “composite behavioral score” vector. This vector served as an individual specific composite marker of behavioral scores, as revealed in the weak off-diagonal values in the covariance matrix of this vector across subjects (Fig. 5*D*, top). dGC and iGC features of individual tasks, as well as a combination of tasks (relational and working memory), were used to then predict the composite score marker for individual subjects, using the same leave-one-out procedure as described above. The observed and predicted set of composite scores was correlated across subjects. The distribution of observed versus predicted correlation values for each subject (Fig. 5*D*, values on main diagonal, bottom, yellow) were compared against between-subject correlation values (Fig. 5*D*, off-diagonal values, bottom, gray) using a Kolmogorov–Smirnov test.

10.1523/ENEURO.0512-19.2019.f5-2 Figure 5-2 Behavioral score predictions based on GC connectivity strengths. ***A***, Correlation between predicted and observed behavior scores based on dGC connectivity strengths. Rows, Task scans from which GC estimates were derived; columns, behavior scores predicted (key, Extended Data Fig. 5-1). Red-blue color scale indexes positive and negative correlations, respectively. Black highlighted squares, Significant *p* values (*p* < 0.05) following Benjamini–Yekutieli correction for multiple comparisons. ***B***, Same as in ***A***, but predictions based on iGC connectivity strengths. ***C***, Same as in ***A***, but predictions based on PC connectivity strengths. ***D***, Same as in Figure 5*D* bottom, but cumulative distributions of correlation coefficients, for composite score predictions based on GC estimates from each task. Other conventions are the same as in Figure 5*D* (main text). Download Figure 5-2, EPS file.

### Data availability

Data used in the study are available in the public domain at the Human Connectome Project database (https://db.humanconnectome.org/). Data-sharing permissions can be found at the HCP website. The code required to replicate results described in the article was developed at the Indian Institute of Science, Bangalore, India, and is freely available online at https://figshare.com/s/9d9131a6780fc8197cf1.

## Results

### GC estimated from slowly sampled fMRI data suffices to distinguish task and resting states

We asked whether iGC and dGC (Extended Data 1 Mathematical Note, Section S1) connectivity would flexibly reconfigure with task demand, by testing whether GC connectivity sufficed to accurately classify among seven different task states or the resting state (Extended Data Fig. 1-1; Materials and Methods; Geweke, 1982, 1984). Data were obtained from 1000 participants from the HCP database (Van Essen et al., 2012). We used connection weights among brain regions in each network (iGC or dGC) as feature vectors in a linear classifier based on SVMs for high-dimensional predictor data. Accuracies for classifying resting state from a working memory task are described first; accuracies for other tasks are presented subsequently.

Both iGC and dGC connectivity were able to distinguish the working memory task from resting state significantly above chance (Fig. 1*B*; *p* < 0.001, permutation test). Maximum median accuracy was 97.3% [95% confidence interval (CI), 96.3–98.0%] with iGC and 92.0% (95% CI, 90.5–93.2%) with dGC (Extended Data Fig. 1-5*B*, Yeo Parcellation; Table 1, a, b; iGC: precision = 97.2, recall = 97.4; dGC: precision = 90.9; recall = 93.2). k-fold (k = 10) cross-validation accuracy was comparable [iGC: median, 97.1% (95% CI, 96.2–97.9%); dGC: median, 91.7% (95% CI, 90.3–93.0%)]. These numbers correspond to maximum cross-validation accuracy across all five parcellations tested (Extended Data Fig. 1-4, Extended Data Fig. 1-5*A*); accuracies with each parcellation are shown in Extended Data Fig. 1-5*B*. Nonlinear classifiers, such as SVMs based on radial basis function kernels produced similar results, with comparably above chance classification accuracy for both iGC and dGC connectivity (Extended Data Fig. 1-5*C*).

**Table 1:** Statistical table

	Figure	Comparison	Type of test	Statistic	Confidence interval or power
a	1*B*	Rest vs working memory best iGC classification accuracy value	Binomial test	Clopper–Pearson confidence intervals	96.3–98.0%
b		Rest vs working memory best dGC classification accuracy value			90.5–93.2%
c		Rest vs task maximum classification accuracy (each bar) vs chance	Permutation test	*p* value	*p* < 0.001
d	1*F*	N-way task classification maximum iGC accuracy value	Binomial test	Clopper–Pearson confidence intervals	73.3–75.4%
e		N-way task classification maximum dGC accuracy value			46.4–48.7%
f		N-way task classification maximum accuracy values (each bar) vs chance	Permutation test	*p* value	*p* < 0.001
g	1*G*	Subtask classification maximum accuracies (each bar) vs chance	Permutation test	*p* value	*p* < 0.05
h	1*H* (left)	Rest vs task dGC classification accuracies with 2×, and 3× sampling rate (vs 1×)	Wilcoxon one-tailed signed-rank	*p* value	2×: *p* = 0.02; 3×: *p* = 0.06
i		Rest vs task iGC classification accuracies with 2×, and 3× sampling rate (vs 1×)			2×: *p* = 0.01; 3×: *p* = 0.01
j	2*C*	ZCA, GEV classification accuracy values with dGC and iGC vs chance	Permutation test	*p* value	*p* < 0.001
k	2*D*	Rest vs task unweighted dGC classification accuracy value (each bar) vs chance	Permutation test	*p* value	*p* < 0.001
l	3*A*	Each dGC, iGC, PC matrix connectivity value bound with black square vs corresponding null distribution	Phase scrambling	*p* value	*p* < 0.05
m	3*C*	Each dGC, iGC, PC matrix connectivity value bound with black square vs corresponding null distribution of phase-scrambled surrogates	Benjamini–Hochberg correction	*p* value	*p* < 0.05
n	5*C*	Each prediction corr value with filled circle in stem plot	Benjamini–Yekutieli correction	*p* value	*p* < 0.05
o	5*D*	Correlation coefficients between observed and predicted composite scores, for the same subject vs across different subjects	Kolmogorov–Smirnov test	*p* value	*p* < 0.001

We repeated these analyses by classifying the six other tasks (Extended Data Fig. 1-1) versus resting state. iGC and dGC connectivity could accurately classify each task from resting state significantly above chance. For iGC, maximum classification accuracies ranged from 90.1%, for emotion versus resting-state classification, to 97.1%, for language versus resting-state classification. Similarly, for dGC, accuracies ranged from 78.1%, for emotion versus resting-state classification, to 92.8%, for language versus resting-state classification (Fig. 1*B*; Table 1, c). In general, classification accuracy increased with more scan timepoints for each task versus resting-state classification (Fig. 1*C*), consistent with GC being an information theoretic measure; we confirmed this result with simulations also (Extended Data Fig. 1-5*D*).

In these analyses, classification accuracies based on dGC were systematically lower than those based on iGC. We asked whether dGC accuracies were poorer due to noise corrupting the fit of the autoregressive model, and whether a more consistent estimate could be obtained by averaging dGC connectivity features, to remove uncorrelated noise, across subjects. We addressed this question by partitioning the data into two groups—a training (T) group and a test (S) groups—with 500 subjects each. We trained the classifier on group T and tested the classifier prediction by averaging GC matrices across several folds of S of size m, each fold containing a few (m = 2, 4, 5, 10, 25, or 50) subjects; the procedure was repeated by exchanging training and test datasets (see Materials and Methods). For the vast majority of tasks (six of seven), the classification accuracy of dGC was >95% with as few as m = 5 subjects within each fold of the test set (Fig. 1*D*). These results suggest that averaging dGC matrices across a few subjects, yielded reliable estimates of dGC connectivity.

We considered other factors that, in addition to intrinsic connectivity differences, could have produced these superior classification accuracies. First, GC-based accuracies for classifying task versus resting-state scans might arise from differences in brain regions activated during each of these scans. In addition to task-relevant sensory input, overt motor responses always occurred during task scans but were absent during resting-state scans (Barch et al., 2013; Glasser et al., 2013). Could GC features discriminate among more subtle connectivity variations across/within tasks? Second, scan data from the HCP database were sampled at a TR of 720 ms, which is considerably faster than the TR for conventional fMRI scans. Would GC accuracies degrade if the data were sampled at a much slower sampling rate (∼2000 ms), which is in line with conventional fMRI TR?

We addressed the first question in two stages. First, we asked whether GC connectivity features would be able to classify which of the seven tasks each subject was performing in the scanner. First, we performed a pairwise classification of each task from the other. Maximum classification accuracies for iGC (dGC) ranged from 87% (67%), for the emotion versus gambling task classification, to 98% (91%), for the language versus social task classification. Again, the number of timepoints for each task proved to be a strong indicator of classification accuracies (Fig. 1*E*): the average intertask classification accuracies were highest for the language task (iGC, 97%; dGC, 88%; *n* = 316 timepoints) and lowest for the emotion task (iGC, 91%; dGC, 77%; *n* = 176 timepoints). Next, we performed an N-way classification analysis across all seven tasks, again using linear SVMs (Materials and Methods). Accuracies were significantly above chance (14.3% for 1-in-7 classification) for classifying among the seven tasks [Fig. 1*F*; maximum accuracy: iGC, 74.4% (73.3 − 75.4%); dGC, 47.6% (46.4 − 48.7%); *p* < 0.001, permutation test; Table 1, d, e, f]. These results indicate that functional connectivity was consistently estimated with GC and reliably different across tasks.

Second, each of the different tasks in the HCP database comprised blocks of contiguous trials, with each block corresponding to one of (at least) two different subtasks (Barch et al., 2013; Extended Data Fig. 1-2). For example, the motor task was composed of blocks of trials involving movements of the right or left hand interleaved with blocks of trials involving movements of the right or left foot. Similarly, the working memory task comprised interleaved blocks of 0-back and 2-back tasks. We asked, therefore, whether GC connectivity could distinguish among subtler variations in brain states across subtasks within each task. We sought to classify across two subtasks for each of six tasks (Extended Data Fig. 1-2). In all cases, except one, both iGC and dGC connectivity discriminated between each pair of subtasks with higher than chance accuracies [Fig. 1*G*; maximum accuracy: iGC, 89.2% (95% CI, 87.6–90.7%); dGC, 80.1% (95% CI, 78.9–82.9%); *p* < 0.05, permutation test; Table 1, g]. These results indicate that GC functional connectivity could accurately distinguish among subtasks within each task as well.

Next, we tested whether GC connectivity estimated from slowly sampled fMRI data could accurately classify task and resting states. We downsampled the data to either one-half (2× TR = 1440 ms) or one-third (3× TR = 2160 ms) of its original sampling rate, by decimation, while also concatenating the decimated data to the end of the subsampled time series to preserve the overall number of timepoints (Materials and Methods). We repeated both of the previous classification analyses—pairwise task versus resting-state classification (Fig. 1*H*, left), as well as N-way intertask classification (Fig. 1*H*, right). Following downsampling, we observed that classification accuracies were marginally higher than accuracies in the original data, in the case of dGC (2×, *p* = 0.02; 3×, *p* = 0.06, Wilcoxon one-tailed signed-rank test; Table 1, h) and were even higher than those in the original data, in the case of iGC (2×, *p* = 0.01; 3×, *p* = 0.01; Table 1, i), across tasks. These results indicate that the superlative sampling rate of the HCP fMRI data was not the primary reason for these high classification accuracies for GC-based classification.

We performed additional control analyses to confirm that these results were not due to data non-stationarity, biases in GC estimation, or head motion artifacts.

As a first control analysis, we repeated the classification analyses including only subjects for whom the data passed tests of stationarity (Materials and Methods; Extended Data Fig. 1-7); typically, data from >99% of subjects passed three of four tests of stationarity (except for the consistency test) across all tasks. Mean GC matrices for each task and resting scan closely resembled those of the population for subjects whose data passed all four tests of stationarity across all tasks (*n* = 141; Extended Data Fig. 1-6*A*). Statistical tests revealed that dGC connectivity was only marginally different for this subset of subjects (proportion of significantly different connections: 6.3 ± 0.9%, mean ± SE, across tasks; Kolmogorov–Smirnov test with Benjamini–Hochberg correction for multiple comparisons), whereas iGC connectivity was substantially different (80.6 ± 8.0%, mean ± SE). Nevertheless, accuracies for classifying task versus resting state, as well as for classifying among tasks, were very similar and, in fact, marginally higher for the subjects who passed tests of stationarity compared with the population (Extended Data Fig. 1-6*B*).

As a second control, we repeated the same analyses by deriving GC estimates with a single full regression (one-stage GC), instead of with separate full and reduced regressions (two-stage GC; Materials and Methods); this analysis was necessary due to recent observations that the two-stage GC model can produce biased estimates, especially with incorrectly specified model orders (Stokes and Purdon, 2017; Barnett et al., 2018). Empirically, GC estimates for each of these methods were numerically different, but tightly correlated across subjects (Extended Data Fig. 1-6*E*) and tasks (Extended Data Fig. 1-6*F*): correlation values range from 0.94 to 0.97 for dGC (*p* < 0.001; Extended Data Fig. 1-6*D*). As before, we observed a very similar pattern of classification accuracies with the single full regression model (N-way classification accuracy among seven tasks computed with the Shirer et al. (2012) 14-network parcellation: 48.3% based on dGC, 56.4% based on iGC) versus when GC was estimated with separate full and reduced regressions (47.6% based on dGC, 56.2% based on iGC; chance accuracy, 14.3% for 1-of-7 classification).

As a third control, we sought to remove the contribution of motion artifacts to these superlative classification accuracies. The minimally preprocessed fMRI data of the HCP are already motion corrected, based on the FSL MCFLIRT algorithm (Van Essen et al., 2012). We further controlled for motion artifacts using “motion scrubbing” (Power et al., 2012) by discarding frames with FD values >0.5 mm (see Materials and Methods). Overall, across all task and resting-state scans <2% of frames were discarded with this approach (Extended Data Fig. 1-6*G*). We recomputed GC values on the motion-scrubbed data, for each of the 1000 subjects (Materials and Methods), and repeated the task-versus-rest and N-way task classification analyses. Classification accuracies following motion scrubbing were closely similar and marginally (albeit significantly) higher than accuracies obtained with the original data (Extended Data Fig. 1-6*C*; *p* < 0.01, one-tailed signed-rank test).

As a fourth control, we sought to test how well the BOLD signal itself would classify among tasks, based on the mean and SD of fMRI time series parcellated with the Shirer et al. (2012) 14-network parcellation (see Materials and Methods). Accuracies for classifying a task state from rest were significantly lower [range, 62.7–67.7%; median, 65.9%) compared with both dGC- and iGC-based classification accuracies (*p* < 0.01, one-tailed signed-rank test). In fact, N-way classification accuracy was 15.7%, only marginally above chance of 14.3%

These results demonstrate that both iGC and dGC yielded task-specific signatures of functional connectivity even with slowly sampled fMRI data (TR, ∼2000 ms): these estimates were consistent across subjects and reliably different across tasks to permit successful classification. Furthermore, these superlative classification accuracies were obtained despite widely held caveats concerning the application of GC to fMRI data (Stokes and Purdon, 2017), suggesting that even if individual fMRI-GC network connections are unreliably estimated for a given task, the difference in fMRI-GC network connectivity across tasks was sufficiently reliable and informative to permit accurate classification among them.

### Correlation-purged GC connectivity suffices for accurate task-state classification

Correlation-based (zero-lag) connectivity measures (e.g., PCs) have been widely applied to estimate functional connectivity from fMRI data (Liang et al., 2012; Ryali et al., 2012). In fact, several previous studies (Smith et al., 2011; Seth et al., 2013) have argued that correlation-based measures are more reliable and should be preferred to lag-based measures like GC (Seth et al., 2015), for estimating functional connectivity with fMRI data. We tested this claim here with a threefold analysis approach.

First, we asked how classification accuracies based on PC connectivity would compare with those reported above, based on GC connectivity. Maximum classification accuracies with PC connectivity ranged from 96% to 99% for task versus resting-state classification and were consistently higher than accuracies with GC connectivity (Fig. 2*A*). These results are along the following expected lines: estimators based on same-time covariance, such as PC, are less susceptible to noise than those based on lagged covariance, such as GC (derived analytically in the Materials and Methods section Functional connectivity estimation and classification with partial correlations). In addition, as mentioned previously, GC is an information theoretic measure: classification accuracy with iGC and dGC increased systematically with more scan timepoints, asymptotically matching PC accuracies (Extended Data Fig. 1-5*D*).

Second, we asked whether lag-based connectivity could accurately classify task from resting state, once the data were purged of all instantaneous correlations. To accomplish this, we adopted the following two approaches: (1) ZCA and (2) GEV decomposition (Materials and Methods). Briefly, ZCA (or the Mahalanobis transformation) produces whitened time series data that are closest, in a least-squares sense, to the original regional time series data. As an alternative approach, we decorrelated both task and resting-state time series jointly by projecting them onto a single set of GEVs. These approaches provided empirical upper and lower bounds on the GC performance on correlation-purged data (Materials and Methods).

GC connectivity features sufficed to successfully classify all tasks from resting state, even in correlation-purged data. With ZCA, iGC accuracies ranged from 84% to 96%, whereas dGC accuracies ranged from 82% to 96% across tasks. With GEVs, iGC accuracies ranged from 60% to 71%, whereas dGC accuracies ranged from 56% to 76% across tasks; in each case, classification accuracies were significantly above chance (*p* < 0.001, permutation test; Table 1, j). We confirmed that performance in each case was not an artifact of the decorrelation procedure (ZCA/GEV) by randomly interchanging task and resting-state labels for each pair of datasets across subjects (Materials and Methods); shuffling labels reduced classification accuracy to chance. Note that in every case, classification performance based on PC connectivity was at chance (Fig. 2*C*), a direct consequence of removing instantaneous correlations from the data. Despite this, classification accuracies based on iGC connectivity were not at chance; in the next section, we discuss potential reasons for these differences between iGC and PC classification accuracies.

Third, we asked whether an unweighted directed graph (digraph) network representation—whose edges indicated the dominant direction, but not the magnitude, of connectivity (Fig. 2*D*)—would suffice to distinguish task from resting brain states (Materials and Methods). Again, dGC directed graphs successfully distinguished each task from resting state well above chance. Classification accuracies ranged from 56% for the motor task versus resting-state classification to 68% for the relational task versus resting state; for each task, classification accuracies were significantly above chance (*p* < 0.001; permutation test; Table 1, k). Interestingly, we did not see a strong influence of the number of data points on classification accuracy in this case (Fig. 2*D*, purple dots). For instance, the emotion task (*n* = 176 timepoints) was classified with an accuracy of 62% from resting state, which was comparable to the classification accuracy of working memory (*n* = 405 timepoints) from resting state (64%). Both iGC and PC, which are symmetric connectivity measures, could provide no directed connectivity information.

These results demonstrate that lag-based connectivity contained sufficient information to classify task from resting state even when instantaneous correlations were entirely purged from the data. Moreover, unweighted directed connectivity graphs alone, indicating the direction, but not scalar magnitude, of GC connectivity, sufficed to accurately classify task from resting brain states. These findings indicate that directed functional connectivity measures, like dGC, provide connectivity information that is distinct from, and complementary to, what can be obtained with undirected functional connectivity measures, like PC.

### Instantaneous and directed GC identify complementary aspects of functional connectivity

What characteristics of functional connectivity are respectively identified by instantaneous and lag-based connectivity? And how can lag-based connectivity be reliably estimated with fMRI data, which is sampled at timescales that are orders of magnitude slower than neural timescales? We addressed both of these questions, first, with simulations (this section) and then, with real data (next section).

First, we tested the ability of GC to reliably recover functional interactions in simple, two-node feedforward networks operating at different timescales (Fig. 3*A*). We simulated fMRI data using a two-stage model (Materials and Methods), as follows: (1) a latent variable model that describes the dynamics of the nodes (vector Ornstein–Uhlenbeck process; Ganguli et al., 2008); and (2) a convolution of these neural dynamics with a hemodynamic response function to obtain the simulated fMRI time series (Smith et al., 2011; Seth et al., 2013). Based on this model, we simulated activity in two two-node networks. In the first network, individual node decay timescales were set to 50 ms, whereas in the second network, these were set to 1000 ms (Extended Data Fig. 3-1*A*, parameters). For convenience, we refer to these two network timescales as “fast” (50 ms) and “slow” (1000 ms). We then varied the sampling interval (T_s_) of the simulated data from 50 to 1450 ms in steps of 100 ms. Connections at both fast and slow timescales were generally discovered by iGC regardless of sampling interval, although connections at slow timescales were less robustly detected than those at fast timescales (Fig. 3*A*). On the other hand, the connection in the fast timescale network was not discovered by dGC when the sampling interval was >50 ms, which is in line with the results of Smith et al. (2011). However, the connection in the slow timescale network was reliably discovered by dGC across a wide range of sampling intervals, up to and exceeding 1000 ms (Table 1, l). In each case, dGC failed to discover the underlying interaction when the sampling interval was much higher than the slowest timescale in each network, consistent with recent theoretical results (Barnett and Seth, 2017). These findings suggest that dGC can detect slow neural processes, which operate at a timescale slower than TR, in fMRI data.

How might such slow timescales, orders of magnitude slower than spike times and membrane time constants, arise in fMRI data? To answer this question, we availed ourselves of established results in random matrix theory. Connectivity in randomly connected excitatory-inhibitory (E-I) networks of neurons can produce slow timescales, without fine-tuning of network parameters (Rajan and Abbott, 2006; Ganguli et al., 2008; Friston et al., 2014). We modeled sparse, random, net excitatory connectivity in a small network of (*N* = 100) neurons with connection parameters drawn from previous studies (Extended Data Fig. 3-1*B*; Gupta et al., 2000; Holmgren et al., 2003; Ganguli et al., 2008). The eigen spectrum of the network revealed that each network exhibited one eigenvalue close to zero, corresponding to a slow timescale (approximately ≥1000 ms; Fig. 3*B*, bottom left); the latter constitutes an emergent timescale associated with the dominant eigenmode that is a property of network connectivity (Materials and Methods).

We modeled nine such networks, organized into three noninteracting clusters (Fig. 3*B*, top right), as follows: (1) a cluster with a purely feedforward connection across two networks; (2) a cluster with recurrent excitatory (E-E) feedback connections among two networks; and (3) a cluster with recurrent E-I feedback connections among two networks. In each case, connectivity across networks was mediated by a small proportion (5%) of neurons in each network (Extended Data Fig. 3-1*B*, parameters). This configuration mimics “small-world” connectivity in brain networks (Bassett and Bullmore, 2006), with locally connected brain regions interacting through sparse, long-range connections (Sporns et al., 2004). The eigenspectra revealed that dynamics in all clusters operated at timescales of ∼6000 ms, comparable to or slower than the individual network timescales (Fig. 3*B*, bottom right). To simulate fMRI data, we averaged the activity across all 100 neurons in each network and convolved it with a canonical HRF. As before, these nine time series were then sampled at various sampling intervals, including a 750 ms interval mimicking the scan TR, and analyzed with GC to detect significant connections. iGC and dGC identified complementary aspects of connectivity with these simulated data (Fig. 3*C*; Table 1, m). iGC robustly identified feedforward and E-E feedback connections. dGC also estimated these connections, albeit with the following differences. First, in the feedforward network dGC occasionally identified a spurious connection, albeit much weaker in magnitude, in the direction opposite to the true connection (Fig. 3*C*, left column, red dashed line). Second, when the E-E feedback connections were precisely balanced in strength (symmetric), dGC also failed to identify the connection reliably (Extended Data Fig. 3-2*A*). Yet, when these connections were of different strengths, dGC reliably identified both connections and their relative strengths (Fig. 3*C*, middle column, red). In contrast, when the connections were of different signs (E-I feedback), dGC robustly identified both connections, whereas iGC failed to reliably detect this connection (Fig. 3*C*, right column, blue). Yet, together, iGC and dGC identified all three connection types reliably.

Next, we compared the efficacy of connectivity estimation with PCs. While PC robustly identified both feedforward and feedback E-E connections (Fig. 3*C*, left and middle columns, black), it, surprisingly, failed to estimate feedback E-I connections, particularly when these were balanced in strength (Fig. 3*C*, right column, black). When the E and I connection strengths were not balanced, but were strongly biased in favor of the E or the I connection, PC estimates varied with the sign of the more dominant connection (Extended Data Fig. 3-2*B*, right top). These results generalize beyond these particular simulations; in the Extended Data 1 Mathematical Note, Sections S2 and S3, we identify, analytically, network configurations for which PC estimates systematically deviate from ground truth connectivity. We generated data with a seven-node network, whose dynamics were described by a multivariate, autoregressive process. We systematically varied the covariance of the residuals of nodes 1 and 2 in the MVAR model (Y), which is a key factor in determining iGC magnitude (Extended Data 1 Mathematical Note, Section S3, Eqs. 11 and 21). Next, we computed the covariance between the residuals (K) in the regression of activities of nodes 1 and 2 against all other nodes (controlling variables), which is a key factor in determining PC magnitude. Although connectivity estimates based on iGC and PC were highly correlated, PC estimates systematically deviated from iGC estimates in value (Extended Data Fig. 3-2*C*, left). In fact, for iGC covariance (Y) values ranging from −0.3 to 0.0, indicating weak inhibitory functional connectivity, PC covariance (K) values were positive, ranging from 0 to 0.3, indicating excitatory functional connectivity (Extended Data 1 Fig. 3-2*C*, right, open squares). For these configurations, therefore, PC connectivity deviated systematically from ground truth. The analytical relationship between PC connectivity and iGC connectivity explains this pattern of systematic deviations (Extended Data 1 Mathematical Note, Section S3, Eq. 23). Briefly, the relationship indicates that PC reflects a mixture of instantaneous and lagged connectivity rather than solely instantaneous interactions. Removing lagged interactions restores the identity between iGC and PC (Extended Data Fig 3-2*C*, right, open circles), as evidenced by setting the coefficients of the AR matrix to zero (Extended Data Mathematical Note, Section S3, Eq. 11). These results highlight caveats with using zero-lag correlation measures, like partial correlations, compared with lag-based measures, like GC, for estimating connectivity with neural time series.

Together, these results indicate that instantaneous and lag-based connectivity measures can reveal complementary aspects of brain connectivity. In addition, the results challenge the notion that correlation-based measures, like PC, should be favored over lag-based measures, like dGC for measuring functional connectivity in the brain (Smith et al., 2011). Rather, the strengths and weaknesses of each measure (GC and PC) must be recognized when seeking to apply these to brain-imaging data.

### Identifying a cognitive core system based on GC connectivity

Our classification analyses and simulations suggested that iGC and dGC reliably recover task-specific brain networks, the latter when slow-timescale processes occur within the network. We asked whether iGC and dGC connectivity merely reflected reliable statistical patterns of brain activity or whether it would be relevant for understanding the nature of information flow in the brain and its relationship to behavior. To answer this question, we investigated whether each measure would identify brain networks with consistent outflow and inflow hubs across tasks.

Before the analysis of real data, we validated RFE by applying it to estimate connectivity differences in two simulated networks (Fig. 4*A*,*B*). RFE accurately identified connections that differed in simulation ground truth: specifically, differences in fast timescale connections were reliably identified by iGC, and in slow timescale connections by dGC (Fig. 4*B*, bottom). We also tested whether dGC and iGC would be able to accurately identify differences in directed information flow among network configurations, even with systematic differences in hemodynamic lags among network nodes. For this, we estimated GC for 100 simulated participants with the same two ground truth network configurations (as shown in Fig. 4*A*), except with the following four different scenarios of hemodynamic lag differences (Materials and Methods): (1) same mean HRF onset (μl = 3 s) across all nodes; (2) source node HRF onset lagging the destination node by 1 s ($\mu_{\text{L-src}}\;>\;\mu_{\text{L-dst}}$); (3) source node HRF onset leading destination node by 1 s ($\mu_{\text{L-src}}\;>\;\mu_{\text{L-dst}}$); and (4) mixed latencies of lead and lag such that 50% of simulated participants had the source node lagging the destination node and vice versa for the remaining 50% simulated participants. We performed these simulations by sampling the onset latency for each participant from a normal distribution, with SDs (σ_L_) ranging from 0 to 1 s (in steps of 0.2 s) across simulations (Extended Data Fig. 3-2*D*,*E*). The relative magnitudes of these HRF onset latency differences, and their SDs, are comparable to their magnitudes observed in human data (Chang et al., 2008). RFE was then used to identify the most discriminative connections between the two networks.

First, we observed that across the different onset latency scenarios, GC connection strength magnitude generally decreased with increasing σ_L_ values (Extended Data Fig. 3-2*D*); an interesting exception was iGC connection strengths when source HRF onset led the destination HRF (case 3, above; Extended Data Fig. 3-2*D*, bottom row, dark blue curves). For subnetwork ABC, with fast (50 ms) timescales, dGC revealed the correct directionality of connectivity (positive ΔdGC; Extended Data Fig. 3-2*D*, bottom row) consistently in only one of the four cases (case 3), when the source node onset systematically led the destination node (Extended Data Fig. 3-2*D*, top row, odd columns, dark blue curves). On the other hand, for subnetwork DEF, with slow (1000 ms) timescales, dGC revealed the correct directionality of connectivity in three of the four cases (Extended Data Fig. 3-2*D*, top row, even columns: dark blue, light blue, and black curves); all except case 2, where the source node onset systematically lagged the destination node (Extended Data Fig. 3-2*D*, top row, red).

In line with these results, RFE with dGC features correctly identified the directionality of the most discriminative connections in no case for the fast subnetwork (Extended Data Fig. 3-2*E*, rows 1–2, ABC subnetwork), but correctly identified the directionality of these connections in three of four cases for the slow subnetwork (Extended Data Fig. 3-2*E*, rows 1–2, DEF subnetwork). RFE with iGC features identified maximally discriminative connections (albeit not their directionality) in all cases (Extended Data Fig. 3-2*E*, rows 3–4). Thus, RFE based on dGC and iGC accurately identified the relevant connections, but not always their directionality, even when systematic variations in hemodynamic lag occurred across regions. Together, these results indicate that fMRI-GC can identify differences in connectivity at slow timescales despite systematic differences and heterogeneity in HRF onset latencies across brain regions.

Next, with the real fMRI (HCP) data, we sought to identify a common core of task-generic connections across cognitive tasks. For this, we applied a feature selection approach—recursive feature elimination (Materials and Methods)—a technique that identifies a minimal set of features that provide maximal cross-validation accuracy (generalization performance; Guyon and Elisseeff, 2003). We applied RFE to classify tasks versus resting state; we chose these six tasks (all tasks except the motor task) as being the most likely to engage common cognitive control mechanisms (Fig. 4*C*). For these RFE analyses, we used a 14-network functional parcellation (Shirer et al., 2012), as it consistently gave good classification accuracies with both iGC and dGC connectivity (Extended Data Fig. 1-5*B*). Following RFE, we applied a binomial test across tasks (Materials and Methods) to identify a common core of task-generic connections, separately for iGC and dGC.

RFE identified distinct task-generic networks with iGC and dGC, which comprised of connections that distinguished a majority of tasks from resting state. The iGC task-generic network revealed a visuospatial network hub, which connected with the anterior salience, dorsal default mode network (DMN), and higher visual and posterior salience networks (Fig. 4*D*, right). The dGC task-generic network confirmed the hub-like connectivity of the visuospatial network but, in addition, revealed consistent directed information outflow from the visuospatial network to the other networks (Fig. 4*D*, left). In addition, dGC revealed consistent inflow into the higher visual network across tasks, including from the visuospatial, right executive control, and auditory networks, consistent with the ability of top-down inputs from these networks to strongly modulate sensory encoding in higher visual cortex (Gilbert and Li, 2013). Finally, the higher visual network projected consistently to the sensorimotor network, suggesting a final common pathway across these tasks for influencing behavior. Interestingly, the only network providing inflow into the visuospatial network hub was the anterior salience network, which is in line with the findings of a previous study that indicated a role for the salience network in controlling other task-positive networks (Sridharan et al., 2008).

Similarly, we asked whether iGC and dGC could identify connections that were maximally discriminative across tasks (task-discriminative networks). Because some network connections may not be present for any task, task-discriminative connections are not simply the complement of the task-generic connections. As before, we repeated the RFE analysis, but this time based on an N-way classification across the six tasks (all except the motor task, Materials and Methods), seeking to identify connections that discriminated each task, from each of the other five tasks (Fig. 4*E*).

This analysis identified iGC and dGC connections among the vast majority of networks as being important for discriminating among tasks. Specifically, with iGC, basal ganglia connectivity was the most task-discriminative, whereas for dGC visuospatial network inflow and language network outflow were among the most discriminative (Fig. 4*F*). Connections with the precuneus were strongly discriminative across both iGC and dGC networks. Notable exceptions to these trends were the sensorimotor network and ventral DMN (vDMN). The sensorimotor network exhibited very few task-discriminative connections based on iGC (1 of 13) and dGC (3 of 26), whereas the vDMN exhibited only 1 of 13 task-discriminative connections based on iGC. We further observed that each task recruited a distributed pattern of connectivity across networks (Materials and Methods), which was sufficiently characteristic of each task to permit accurate classification (Extended Data Fig. 4-2*A*). We also correlated the β weights of the 11 overlapping connections across iGC and dGC and found no significant correlations (*r* = −0.18, *p* = 0.59). The results indicate that the task-discriminative information flow patterns, as measured by iGC or dGC connectivity, arise from distinct, distributed networks across the entire brain.

We also tested whether PC would identify task-generic and task-discriminative connections that were more in line with those identified by iGC or dGC or both (Extended Data Fig. 4-2*B–D*). Both task-generic and task-discriminative connections identified with PC revealed significant overlap with both iGC (task-generic, 75% overlap; task-discriminative, 65.2% overlap; *p* < 0.05, randomization test) and dGC (task-generic, 100% overlap; task-discriminative, 78.3% overlap; *p* < 0.05). These findings are consistent with our theoretical result that PC connectivity reflects a mixture of iGC and dGC connectivity.

### Predicting behavioral scores with GC connectivity

To address the relevance of GC for understanding brain–behavior relationships, we tested whether the strength of functional connections estimated with iGC and dGC could predict interindividual variations in behavioral scores as measured by a standard cognitive battery (Materials and Methods; Extended Data Fig. 5-1).We used a leave-one-out prediction analysis based on multilinear regression followed by robust correlations of predicted and observed scores [Fig. 5*A*; *p* < 0.05 with Benjamini–Yekutieli (BY) correction; Materials and Methods].

Both iGC and dGC predicted key behavioral scores (Fig. 5*C*; Table 1, n). Several scores were predicted uniformly well by iGC across tasks (Fig. 5*B*, right, Fig. 5*C*, bottom, Extended Data Fig. 5-2*B*). Scores of fluid intelligence (Penn progressive matrices), spatial orientation (Penn line orientation test), grip strength, endurance, and language (picture-vocabulary and reading; Fig. 5*B*, right), were all well predicted by iGC (Extended Data Fig. 5-2*B*; *r* = 0.104–0.363; *p* < 0.01). On the other hand, dGC-based predictions were more selective, in that several behavioral scores were best predicted by dGC based on specific tasks alone (Fig. 5*B*, left, Fig. 5*C*, top, Extended Data Fig. 5-2*A*). For instance, dGC in the gambling task alone predicted self-reported scores of fear (*r* = 0.139, *p* < 0.001), and dGC in the motor task alone predicted median reaction time in the fluid intelligence test (*r* = 0.123, *p* < 0.001) and self-reported scores of perceived emotional support (*r* = 0.113, *p* < 0.001). In addition, dGC in the working memory task predicted a range of scores in the “cognition” category, including list sorting (Fig. 5*B*, left, pink; *r* = 0.119, *p* = 0.000), fluid intelligence, and picture discrimination speed (Fig. 5*C*, top, Extended Data Fig. 5-2*A*).

Similarly, we used PC functional connection strengths as features for predicting interindividual differences in behavioral scores. We observed that 129 behavioral scores were successfully predicted based on PC connectivity (Extended Data Fig. 5-2*C*, following BY correction for multiple comparisons), compared with 39 scores based on dGC connectivity (Extended Data Fig. 5-2*A*) and 92 scores based in iGC connectivity (Extended Data Fig. 5-2*B*). Approximately 54% of the scores predicted well by PC (70 of 129) were also predicted well by either dGC or iGC. On the other hand, behavioral scores that were predicted well by PC, but not by GC, included reaction times in the Penn word memory test and Penn emotion recognition test, as well as several scores of the language task (Extended Data Fig. 5-2*C*).

Next, we compared the connection features that led to successful predictions based on GC and PC. For this, we *z*-scored the connection strengths (individually) and repeated the prediction process (Materials and Methods) separately with dGC features, iGC features, and PC features derived from each of the seven tasks. Seventeen of these predictions were significant (following BY correction) across all three connectivity features (Extended Data Fig. 5-2*A–C*). We then correlated the β weights for each entry of the iGC matrix with those of the PC matrix across these 17 predictions. For dGC, the upper and lower triangular portions of the matrix were correlated separately, with the corresponding PC connection weights. We then computed the mean correlation (*r*) values across all 91 features (iGC vs PC) and 182 features (dGC lower and upper matrix vs PC).

We observed an interesting dissociation among PC, iGC, and dGC. Connection features that were relevant for behavioral predictions with PC overlapped highly with iGC features, but not with dGC features (PC vs iGC: *r* = 0.39 ± 0.02, mean ± SD; PC vs dGC: *r* = 0.03 ± 0.02; *p* < 0.001, rank sum test). The results provide further empirical evidence for a clear distinction between connectivity computed with instantaneous (PC, iGC) and lag-based (dGC) measures.

Finally, we tested whether GC connectivity could predict a combined set of behavioral scores unique to each subject. For this, we created a vector of all independent behavioral scores (composite score; Materials and Methods) and confirmed that this composite behavioral score uniquely identified each subject in the database, as evidenced by the highest values along the main diagonal of the intersubject correlation matrix (Fig. 5*D*, top). Following this, we performed the leave-one-out prediction, as before, except that we used dGC and iGC connectivity features from two of the tasks alone (working memory and relational; also see Extended Data Fig. 5-2*D*). We then tested whether each subject’s predicted composite score would correlate best with her/his own observed composite scores. Although we did not observe the highest correlation values consistently along the main diagonal, the distribution of correlation coefficients along the diagonal were significantly different (and higher) than the distribution of off-diagonal correlation coefficients (Fig. 5*D*, bottom; *p* < 10^−15^, Kolmogorov–Smirnov test; Table 1, o). Interindividual variation GC connectivity, therefore, contained sufficient information to accurately identify subject-specific behavioral scores in this cohort of subjects.

In summary, the ability to successfully predict subject-specific behavioral scores suggests that GC functional connectivity is relevant for understanding brain–behavior relationships. Moreover, connection features that were relevant for behavioral predictions with PC overlapped highly with iGC, but not with dGC, thereby validating our simulation results regarding the complementarity of iGC and dGC connectivity estimates.

## Discussion

Neural processes in the brain range from timescales of milliseconds, for extremely rapid processes (e.g., sound localization), to timescales of several seconds to minutes, for processes that require coordination across diverse brain networks (e.g., when having a conversation), and to timescales of hours to days for processes that involve large-scale neuroplastic changes (e.g., when learning a new language). Coordinated activity among brain regions that mediate each of these cognitive processes should manifest in the form of functional connectivity among these regions at the corresponding timescales. Our results indicate that applying GC with fMRI data permits estimating behaviorally relevant functional connectivity at a timescale corresponding to the sampling rate of fMRI data (seconds).

The application of GC to neuroscience is a contentious topic, for a variety of reasons (Chang et al., 2008; Friston et al., 2013; Seth et al., 2013; Wen et al., 2013; Stokes and Purdon, 2017). One particular challenge stems from the use of the word “causality”: the notion of causality in GC is different from the notion of interventional causality (Pearl, 2011). Our use of the term Granger causality here purely reflects its application as a marker of information flow among brain networks (Roebroeck et al., 2005; Seth et al., 2013) and is not meant to indicate causality in a physical, interventional sense.

With this understanding, our results contain three key insights. First, we show that either iGC or dGC connectivity suffices to reliably classify task-specific cognitive states with superlative accuracy (Fig. 1*B*). Instantaneous GC and directed GC—both measures of conditional linear dependence and feedback (Geweke, 1984)—were able to robustly estimate task-specific functional interactions even with slowly sampled fMRI data. Our simulations suggest that GC connectivity is relevant for estimating slow, emergent interactions among brain networks (Chang et al., 2008; Smith et al., 2011; Friston et al., 2013; Seth et al., 2013; Wen et al., 2013).

Second, we show that functional connections identified by iGC and dGC carry complementary information, both in simulated and in real fMRI recordings, and we demonstrate key caveats with using correlation-based measures of functional connectivity like partial correlations, despite superior classification accuracies with these latter measures. First, PC fails to correctly infer reciprocal excitatory–inhibitory interactions, which can be accurately inferred with lag-based methods like dGC. Second, PC may yield incorrect estimates of functional connectivity that do not match ground truth (Extended Data Fig. 3-2*C*). In particular, when the data are well described by an autoregressive model framework, our results suggest that instantaneous connectivity measures, like iGC, provide more accurate descriptions of functional connectivity than PC. Third, even with data completely purged of partial correlations, dGC connectivity was sufficient to classify task-specific cognitive states (Fig. 2*C*). In fact, unweighted directed connectivity alone sufficed to produce accurate classification at accuracies significantly above chance (Fig. 2*D*). These results indicate that information flow mapped by GC connectivity can be complementary to that of PC, and highlights the need for examining diverse measures, both instantaneous and lag based, to obtain a complete picture of functional connectivity in the brain.

Finally, differences in interindividual iGC and dGC connectivity were able to explain interindividual variation in behavioral scores on various cognitive tasks and to identify an individual-specific composite marker of behavioral scores with high accuracy. Perhaps because these behavioral scores were acquired in a separate testing session outside the scanning session (Barch et al., 2013), the effect sizes were small (albeit significant) and were comparable to effect sizes in previous studies using large, heterogeneous subject cohorts (Smith and Nichols, 2018). Nevertheless, the results suggest that GC connectivity was both individual specific and stable over timescales exceeding the scan session to permit accurate prediction. Moreover, in our analysis, each subject’s behavioral score was predicted based on GC connectivity, whereas the regression β weights—representing the relationship between GC connectivity and behavior—were computed from the population of all subjects excluding that subject (Fig. 5*A*). Successful predictions, therefore, indicate a consistent mapping between GC connectivity and behavioral scores across the population of subjects. These findings complement recent results showing that dynamic resting-state functional connectivity, based on correlations, can explain significant variance in human behavioral data (Liégeois et al., 2019) and indicate the relevance of lag-based connectivity measures for understanding brain–behavior relationships.

Does the GC discriminatory power rely on directed functional connectivity in the underlying neural response or on systematic distortions of this connectivity induced by subsampling (Seth et al., 2013) and hemodynamic filtering (Lin et al., 2009; Solo et al., 2018)? While our findings cannot completely rule out the latter hypothesis, we next address three key caveats raised by previous studies for estimating functional connectivity with fMRI-GC and argue why our results support the former hypothesis.

First, several studies have shown that subsampling of neural time series, at the scale of fMRI TR, renders functional connections undetectable with GC (Lin et al., 2009; Smith et al., 2011; Seth et al., 2013, 2015). In these studies, GC was estimated with simulated fMRI time series, sampled at an interval (TR) of seconds, and failed to recover underlying neural interactions, which occur at millisecond timescales (Smith et al., 2011). However, these claims depended strongly on the nature and timescale of the connectivity in the networks used in these simulations. For instance, a widely cited study (Smith et al., 2011) used purely feedforward connectivity matrices with a 50 ms neural timescale in their simulations, and argued that functional connections are not reliably inferred with GC applied to simulated fMRI data. In addition to being neurally implausible, such purely feedforward network configurations yield eigenmodes whose slowest timescales are identical to the timescales of individual nodes (Sundaresan et al., 2017). Therefore, such a configuration rendered lag-based measures, like GC, irrelevant for estimating neural interactions from slowly sampled fMRI data (Smith et al., 2011; Seth et al., 2013). Furthermore, such connectivity precludes the occurrence of slower, behaviorally relevant timescales of seconds, which readily emerge in the presence of feedback connections, both in simulations (Rajan and Abbott, 2006; Ganguli et al., 2008) and in the real brain (Friston et al., 2014; Runyan et al., 2017; Vidaurre et al., 2017). Our simulations show that slow-timescale interactions emerge in networks with sparse, random, net excitatory connectivity, mimicking connectivity in the neocortex (Gupta et al., 2000; Holmgren et al., 2003; Ganguli et al., 2008). While earlier studies have used large-scale, biologically plausible models (Deco et al., 2009; Krishnan et al., 2018) to demonstrate the emergence of slow (<0.1 Hz) emergent functional interactions among brain networks, our results build on these previous findings and show that such emergent, functional interactions at slow timescales can be readily inferred from simulated fMRI data with GC. In fact, GC connectivity continued to robustly classify distinct task states even when data were sampled at 2× or 3× the original sampling interval of the fMRI data. Thus, while it is likely that GC applied to fMRI data is unable to detect connections at timescales faster than TR, our results show that sufficient distinguishing information occurs in slow-timescale connections to enable accurate intertask classification with fMRI-GC. Subsampling alone may also produce spurious GC causality. The precise conditions under which spurious GC arises for continuous time vector autoregressive processes, possibly with time delay in between the nodes, is an area of active research, and must be addressed in future studies (Lin et al., 2009; Barnett and Seth, 2017).

Second, previous studies have shown that systematic differences in hemodynamic (HRF) lags (e.g., time to onset or time to peak) among brain regions may produce spurious dGC estimates (Friston, 2009; Seth et al., 2013; Solo et al., 2018). With simulations, we demonstrated that fMRI-GC could identify differences in slow-timescale network connectivity, despite systematic differences and heterogeneity in HRF onset latencies across nodes (Extended Data Fig. 3-2*D*,*E*). In all cases, applying recursive feature elimination with either dGC or iGC features identified the precise subset of connections that distinguished distinct network configurations. In a majority of cases, dGC also correctly identified the directionality of these connections. In our simulations, the only scenario in which dGC features failed to identify the directionality of connections correctly was when the onset latency in the “destination” nodes was biased to be systematically earlier than those in the “source” nodes. Nevertheless, in the real data it is unlikely that systematic inter-regional HRF differences contributed to the observed superior classification accuracies. Variations in HRF delays would indeed confound dGC connectivity estimates—if they occurred consistently between brain regions across subjects and tasks (Extended Data Fig. 3-2*D*, red curves). Yet, such a scenario cannot account for the high classification accuracies among tasks and subtasks based on dGC connectivity alone. In other words, even if HRF latency differences did systematically bias dGC connectivity estimates, these estimates were sufficiently and reliably different across task cognitive states to permit accurate classification among them. To our knowledge, our study provides the first direct experimental validation of the ability of GC networks to distinguish cognitive states as a marker of their potential utility for identifying these states. Moreover, network properties of key regions identified with fMRI-GC were consistent with their known functional properties of these regions. For instance, dGC identified the visuospatial network as an information outflow hub across all six cognitive tasks (Fig. 4*D*, left). The visuospatial network comprises frontal cortex regions, including the frontal eye field as well as the posterior parietal cortex, which are both widely implicated in visuospatial attention control (Corbetta et al., 1998; Behrmann et al., 2004; Schall, 2004; Thompson and Bichot, 2004). In addition, the only network that provided task-generic incoming connections to the visuospatial network was the anterior salience network comprising the anterior frontoinsular cortex and the anterior cingulate cortex (Dosenbach et al., 2008; Chen et al., 2013), regions implicated in feature-based attention and the suppression of distractors (Li et al., 2018). Information outflow from these key networks identified by dGC is consistent with their role in attention and executive control.

Third, simulations and theoretical results indicate that scanner noise can degrade or even obliterate GC connectivity estimates (Seth et al., 2013). On the other hand, our classification accuracies suggest that GC estimates were sufficiently robust to scanner noise to permit accurate task and subtask classification in these data. In fact, we show that averaging dGC connectivity across the data of as few as five subjects improves classification accuracy to >95% for nearly all tasks (Fig. 1*D*). Such superlative classification accuracies are unlikely to have occurred if scanner noise were to significantly degrade GC estimates.

In sum, these results suggest that lag-based methods like GC, applied to fMRI data, can be used infer slow functional interactions in the brain. While the directionality of interactions measured by GC may need to be interpreted with care (Seth et al., 2015; Solo et al., 2018), our results suggest that fMRI-GC may be useful for formulating hypothesis about the role of particular brain regions in providing “top-down” control signals, for modulating activity in other brain regions (Sridharan et al., 2008; Ryali et al., 2011), as well as for investigating the nature of information flow in cortical microcircuits with slow sampling rate techniques, such as calcium imaging (Fallani et al., 2015).The causal role of these brain regions in behavior can then be directly tested with interventional approaches such as transcranial magnetic stimulation or optogenetic inactivation, or by examining patient populations with lesions in specific brain regions (Gaillard et al., 2006). Such a systematic analysis will pave the way for a mechanistic understanding of how flexible functional interactions among brain regions mediate complex cognitive behaviors.

## Synthesis

Reviewing Editor: Bradley Postle, University of Wisconsin Decisions are customarily a result of the Reviewing Editor and the peer reviewers coming together and discussing their recommendations until a consensus is reached. When revisions are invited, a fact-based synthesis statement explaining their decision and outlining what is needed to prepare a revision will be listed below.

The manuscript was accepted after editorial review.

## References

[B1] Arbabshirani MR, Plis S, Sui J, Calhoun VD (2017) Single subject prediction of brain disorders in neuroimaging: promises and pitfalls. Neuroimage 145:137–165. 10.1016/j.neuroimage.2016.02.079 27012503 PMC5031516

[B2] Barch DM, Burgess GC, Harms MP, Petersen SE, Schlaggar BL, Corbetta M, Glasser MF, Curtiss S, Dixit S, Feldt C, Nolan D, Bryant E, Hartley T, Footer O, Bjork JM, Poldrack R, Smith S, Johansen-Berg H, Snyder AZ, Van Essen DC (2013) Function in the human connectome: task-fMRI and individual differences in behavior. Neuroimage 80:169–189. 10.1016/j.neuroimage.2013.05.033 23684877 PMC4011498

[B3] Barnett L, Seth AK (2014) The MVGC multivariate Granger causality toolbox: a new approach to Granger-causal inference. J Neurosci Methods 223:50–68. 10.1016/j.jneumeth.2013.10.018 24200508

[B4] Barnett L, Seth AK (2017) Detectability of Granger causality for subsampled continuous-time neurophysiological processes. J Neurosci Methods 275:93–121. 10.1016/j.jneumeth.2016.10.016 27826091

[B5] Barnett L, Barrett AB, Seth AK (2018) Misunderstandings regarding the application of Granger causality in neuroscience. Proc Natl Acad Sci U S A 115:E6676–E6677. 10.1073/pnas.1714497115 29991604 PMC6055162

[B6] Bassett DS, Bullmore E (2006) Small-world brain networks. Neuroscientist 12:512–523. 10.1177/1073858406293182 17079517

[B7] Bastos AM, Vezoli J, Bosman CA, Schoffelen J-M, Oostenveld R, Dowdall JR, De Weerd P, Kennedy H, Fries P (2015) Visual areas exert feedforward and feedback influences through distinct frequency channels. Neuron 85:390–401. 10.1016/j.neuron.2014.12.018 25556836

[B8] Behrmann M, Geng JJ, Shomstein S (2004) Parietal cortex and attention. Curr Opin Neurobiol 14:212–217. 10.1016/j.conb.2004.03.012 15082327

[B9] Buckner RL, Sepulcre J, Talukdar T, Krienen FM, Liu H, Hedden T, Andrews-Hanna JR, Sperling RA, Johnson KA (2009) Cortical hubs revealed by intrinsic functional connectivity: mapping, assessment of stability, and relation to Alzheimer’s disease. J Neurosci 29:1860–1873. 10.1523/JNEUROSCI.5062-08.2009 19211893 PMC2750039

[B10] Chang C, Thomason ME, Glover GH (2008) Mapping and correction of vascular hemodynamic latency in the BOLD signal. Neuroimage 43:90–102. 10.1016/j.neuroimage.2008.06.030 18656545 PMC2587338

[B11] Chen AC, Oathes DJ, Chang C, Bradley T, Zhou Z-W, Williams LM, Glover GH, Deisseroth K, Etkin A (2013) Causal interactions between fronto-parietal central executive and default-mode networks in humans. Proc Natl Acad Sci U S A 110:19944–19949. 10.1073/pnas.1311772110 24248372 PMC3856839

[B12] Corbetta M, Akbudak E, Conturo TE, Snyder AZ, Ollinger JM, Drury HA, Linenweber MR, Petersen SE, Raichle ME, Van Essen DC, Shulman GL (1998) A common network of functional areas for attention and eye movements. Neuron 21:761–773. 10.1016/S0896-6273(00)80593-0 9808463

[B13] David O, Guillemain I, Saillet S, Reyt S, Deransart C, Segebarth C, Depaulis A (2008) Identifying neural drivers with functional MRI: an electrophysiological validation. PLoS Biol 6:2683–2697. 10.1371/journal.pbio.0060315 19108604 PMC2605917

[B14] Deco G, Jirsa V, McIntosh AR, Sporns O, Kötter R (2009) Key role of coupling, delay, and noise in resting brain fluctuations. Proc Natl Acad Sci U S A 106:10302–10307. 10.1073/pnas.0901831106 19497858 PMC2690605

[B15] De Martino F, Valente G, Staeren N, Ashburner J, Goebel R, Formisano E (2008) Combining multivariate voxel selection and support vector machines for mapping and classification of fMRI spatial patterns. Neuroimage 43:44–58. 18672070 10.1016/j.neuroimage.2008.06.037

[B16] Dhamala M, Rangarajan G, Ding M (2008) Analyzing information flow in brain networks with nonparametric Granger causality. NeuroImage 41:354–362. 10.1016/j.neuroimage.2008.02.020 18394927 PMC2685256

[B17] Ding M, Wang C (2014) Analyzing MEG data with Granger causality: promises and pitfalls In: Magnetoencephalography: from signals to dynamic cortical networks (Supek S, Aine CJ, eds), pp 309–318. Berlin, Heidelberg: Springer.

[B18] Dosenbach NUF, Fair DA, Cohen AL, Schlaggar BL, Petersen SE (2008) A dual-networks architecture of top-down control. Trends Cogn Sci 12:99–105. 10.1016/j.tics.2008.01.001 18262825 PMC3632449

[B19] Fallani FDV, Corazzol M, Sternberg JR, Wyart C, Chavez M (2015) Hierarchy of neural organization in the embryonic spinal cord: Granger-causality graph analysis of in vivo calcium imaging data. IEEE Trans Neural Syst Rehabil Eng 23:333–341. 10.1109/TNSRE.2014.2341632 25122836

[B20] Fox MD, Snyder AZ, Vincent JL, Corbetta M, Van Essen DC, Raichle ME (2005) From the cover: the human brain is intrinsically organized into dynamic, anticorrelated functional networks. Proc Natl Acad Sci U S A 102:9673–9678. 10.1073/pnas.0504136102 15976020 PMC1157105

[B21] Friston K (2009) Causal modelling and brain connectivity in functional magnetic resonance imaging. PLoS Biol 7:e1000033 10.1371/journal.pbio.1000033 PMC2642881 19226186

[B22] Friston K, Moran R, Seth AK (2013) Analysing connectivity with Granger causality and dynamic causal modelling. Curr Opin Neurobiol 23:172–178. 10.1016/j.conb.2012.11.010 23265964 PMC3925802

[B23] Friston KJ, Kahan J, Razi A, Stephan KE, Sporns O (2014) On nodes and modes in resting state fMRI. Neuroimage 99:533–547. 10.1016/j.neuroimage.2014.05.056 24862075 PMC4121089

[B24] Gaillard R, Naccache L, Pinel P, Clémenceau S, Volle E, Hasboun D, Dupont S, Baulac M, Dehaene S, Adam C, Cohen L (2006) Direct intracranial, fMRI, and lesion evidence for the causal role of left inferotemporal cortex in reading. Neuron 50:191–204. 10.1016/j.neuron.2006.03.031 16630832

[B25] Ganguli S, Bisley JW, Roitman JD, Shadlen MN, Goldberg ME, Miller KD (2008) One-dimensional dynamics of attention and decision making in LIP. Neuron 58:15–25. 10.1016/j.neuron.2008.01.038 18400159 PMC7204626

[B26] Gershon RC, Wagster MV, Hendrie HC, Fox NA, Cook KF, Nowinski CJ (2013) NIH toolbox for assessment of neurological and behavioral function. Neurology 80 [11 Suppl 3]:S2–S6. 10.1212/WNL.0b013e3182872e5f 23479538 PMC3662335

[B27] Geweke J (1982) Measurement of linear dependence and feedback between multiple time series. J Am Stat Assoc 77:304–313. 10.1080/01621459.1982.10477803

[B28] Geweke JF (1984) Measures of conditional linear dependence and feedback between time series. J Am Stat Assoc 79:907–915. 10.1080/01621459.1984.10477110

[B29] Gilbert CD, Li W (2013) Top-down influences on visual processing. Nat Rev Neurosci 14:350–363. 10.1038/nrn3476 23595013 PMC3864796

[B30] Glasser MF, Sotiropoulos SN, Wilson JA, Coalson TS, Fischl B, Andersson JL, Xu J, Jbabdi S, Webster M, Polimeni JR, Van Essen DC, Jenkinson M (2013) The minimal preprocessing pipelines for the Human Connectome Project. Neuroimage 80:105–124. 10.1016/j.neuroimage.2013.04.127 23668970 PMC3720813

[B31] Gupta A, Wang Y, Markram H (2000) Organizing principles for a diversity of GABAergic interneurons and synapses in the neocortex. Science 287:273–278. 10634775 10.1126/science.287.5451.273

[B32] Guyon I, Elisseeff A (2003) An introduction to variable and feature selection. J Mach Learn Res 3:1157–1182.

[B33] Holmgren C, Harkany T, Svennenfors B, Zilberter Y (2003) Pyramidal cell communication within local networks in layer 2/3 of rat neocortex. J Physiol 551:139–153. 10.1113/jphysiol.2003.044784 12813147 PMC2343144

[B34] Karampatziakis N, Mineiro P (2014) Discriminative features via generalized eigenvectors In: Proceedings of the 31st International Conference on Machine Learning. PMLR 32:494–502.

[B35] Kessy A, Lewin A, Strimmer K (2018) Optimal whitening and decorrelation. Am Stat 72:309–314.

[B36] Knösche T, Tittgemeyer M (2011) The role of long-range connectivity for the characterization of the functional–anatomical organization of the cortex. Front Syst Neurosci 5:58. 10.3389/fnsys.2011.00058 21779237 PMC3133730

[B37] Krishnan GP, González OC, Bazhenov M (2018) Origin of slow spontaneous resting-state neuronal fluctuations in brain networks. Proc Natl Acad Sci U S A 115:6858–6863. 10.1073/pnas.1715841115 29884650 PMC6042137

[B38] Li V, Michael E, Balaguer J, Castañón SH, Summerfield C (2018) Gain control explains the effect of distraction in human perceptual, cognitive, and economic decision making. Proc Natl Acad Sci U S A 115:E8825–E8834. 10.1073/pnas.1805224115 30166448 PMC6156680

[B39] Liang X, Wang J, Yan C, Shu N, Xu K, Gong G, He Y (2012) Effects of different correlation metrics and preprocessing factors on small-world brain functional networks: a resting-state functional MRI study. PLoS One 7:e32766 10.1371/journal.pone.0032766 22412922 PMC3295769

[B40] Liégeois R, Li J, Kong R, Orban C, Van De Ville D, Ge T, Sabuncu MR, Yeo BTT (2019) Resting brain dynamics at different timescales capture distinct aspects of human behavior. Nat Commun 10:2317. 10.1038/s41467-019-10317-7 31127095 PMC6534566

[B41] Lin F-H, Hara K, Solo V, Vangel M, Belliveau JW, Stufflebeam SM, Hämäläinen MS (2009) Dynamic Granger-Geweke causality modeling with application to interictal spike propagation. Hum Brain Mapp 30:1877–1886. 10.1002/hbm.20772 19378280 PMC2825391

[B42] Marrelec G, Krainik A, Duffau H, Pélégrini-Issac M, Lehéricy S, Doyon J, Benali H (2006) Partial correlation for functional brain interactivity investigation in functional MRI. Neuroimage 32:228–237. 10.1016/j.neuroimage.2005.12.057 16777436

[B43] Nolte G, Ziehe A, Nikulin VV, Schlögl A, Krämer N, Brismar T, Müller K-R (2008) Robustly estimating the flow direction of information in complex physical systems. Phys Rev Lett 100:234101. 10.1103/PhysRevLett.100.234101 18643502

[B44] Ojala M, Garriga GC (2010) Permutation tests for studying classifier performance. J Mach Learn Res 11:1833–1863.

[B45] Pearl J (2011) Causality: models, reasoning, and inference, Ed 2. New York: Cambridge UP.

[B46] Penny W, Friston K, Ashburner J, Kiebel S, Nichols T (2007) Statistical parametric mapping: the analysis of functional brain images. New York: Academic.

[B47] Power JD, Barnes KA, Snyder AZ, Schlaggar BL, Petersen SE (2012) Spurious but systematic correlations in functional connectivity MRI networks arise from subject motion. Neuroimage 59:2142–2154. 10.1016/j.neuroimage.2011.10.018 22019881 PMC3254728

[B48] Rajan K, Abbott LF (2006) Eigenvalue spectra of random matrices for neural networks. Phys Rev Lett 97:188104 10.1103/PhysRevLett.97.188104 17155583

[B49] Roebroeck A, Formisano E, Goebel R (2005) Mapping directed influence over the brain using Granger causality and fMRI. Neuroimage 25:230–242. 10.1016/j.neuroimage.2004.11.017 15734358

[B50] Runyan CA, Piasini E, Panzeri S, Harvey CD (2017) Distinct timescales of population coding across cortex. Nature 548:92–96. 10.1038/nature23020 28723889 PMC5859334

[B51] Ryali S, Supekar K, Chen T, Menon V (2011) Multivariate dynamical systems models for estimating causal interactions in fMRI. Neuroimage 54:807–823. 10.1016/j.neuroimage.2010.09.052 20884354 PMC2997172

[B52] Ryali S, Chen T, Supekar K, Menon V (2012) Estimation of functional connectivity in fMRI data using stability selection-based sparse partial correlation with elastic net penalty. Neuroimage 59:3852–3861. 10.1016/j.neuroimage.2011.11.054 22155039 PMC3288428

[B53] Schall JD (2004) On the role of frontal eye field in guiding attention and saccades. Vision Res 44:1453–1467. 10.1016/j.visres.2003.10.025 15066404

[B54] Seth AK, Chorley P, Barnett LC (2013) Granger causality analysis of fMRI BOLD signals is invariant to hemodynamic convolution but not downsampling. Neuroimage 65:540–555. 10.1016/j.neuroimage.2012.09.049 23036449

[B55] Seth AK, Barrett AB, Barnett L (2015) Granger causality analysis in neuroscience and neuroimaging. J Neurosci 35:3293–3297. 10.1523/JNEUROSCI.4399-14.2015 25716830 PMC4339347

[B56] Shirer WR, Ryali S, Rykhlevskaia E, Menon V, Greicius MD (2012) Decoding subject-driven cognitive states with whole-brain connectivity patterns. Cereb Cortex 22:158–165. 10.1093/cercor/bhr099 21616982 PMC3236795

[B57] Smith SM, Nichols TE (2018) Statistical challenges in “big data” human neuroimaging. Neuron 97:263–268. 10.1016/j.neuron.2017.12.018 29346749

[B58] Smith SM, Miller KL, Salimi-Khorshidi G, Webster M, Beckmann CF, Nichols TE, Ramsey JD, Woolrich MW (2011) Network modelling methods for fMRI. Neuroimage 54:875–891. 10.1016/j.neuroimage.2010.08.063 20817103

[B59] Smith SM, Bandettini PA, Miller KL, Behrens TEJ, Friston KJ, David O, Liu T, Woolrich MW, Nichols TE (2012) The danger of systematic bias in group-level FMRI-lag-based causality estimation. Neuroimage 59:1228–1229. 10.1016/j.neuroimage.2011.08.015 21867760

[B60] Solo V (2016) State-space analysis of Granger-Geweke causality measures with application to fMRI. Neural Comput 28:914–949. 10.1162/NECO_a_00828 26942749 PMC5572774

[B61] Solo V, Poline J-B, Lindquist MA, Simpson SL, Bowman FD, Chung MK, Cassidy B (2018) Connectivity in fMRI: blind spots and breakthroughs. IEEE Trans Med Imaging 37:1537–1550. 10.1109/TMI.2018.2831261 29969406 PMC6291757

[B62] Sporns O, Chialvo D, Kaiser M, Hilgetag C (2004) Organization, development and function of complex brain networks. Trends Cogn Sci 8:418–425. 10.1016/j.tics.2004.07.008 15350243

[B63] Sridharan D, Levitin DJ, Menon V (2008) A critical role for the right fronto-insular cortex in switching between central-executive and default-mode networks. Proc Natl Acad Sci U S A 105:12569–12574. 10.1073/pnas.0800005105 18723676 PMC2527952

[B64] Stokes PA, Purdon PL (2017) A study of problems encountered in Granger causality analysis from a neuroscience perspective. Proc Natl Acad Sci U S A 114:E7063–E7072. 10.1073/pnas.1704663114 28778996 PMC5576801

[B65] Sundaresan M, Nabeel A, Sridharan D (2017) Mapping distinct timescales of functional interactions among brain networks In Proceedings of the 31st Annual Conference on Neural Information Processing Systems (von Luxburg U, Guyon I, Bengio S, Wallach H, Fergus R, Vishwanathan SVN, Garnett R eds), pp 4109–4118 NY: Curran Associates.

[B66] Tavor I, Jones OP, Mars RB, Smith SM, Behrens TE, Jbabdi S (2016) Task-free MRI predicts individual differences in brain activity during task performance. Science 352:216–220. 10.1126/science.aad8127 27124457 PMC6309730

[B67] Thomas Yeo BT, Krienen FM, Sepulcre J, Sabuncu MR, Lashkari D, Hollinshead M, Roffman JL, Smoller JW, Zöllei L, Polimeni JR, Fischl B, Liu H, Buckner RL (2011) The organization of the human cerebral cortex estimated by intrinsic functional connectivity. J Neurophysiol 106:1125–1165. 10.1152/jn.00338.2011 21653723 PMC3174820

[B68] Thompson KG, Bichot NP (2004) A visual salience map in the primate frontal eye field. Prog Brain Res 147:251–262. 10.1016/S0079-6123(04)47019-8 15581711

[B69] Van Essen DC, Ugurbil K, Auerbach E, Barch D, Behrens TEJ, Bucholz R, Chang A, Chen L, Corbetta M, Curtiss SW, Della Penna S, Feinberg D, Glasser MF, Harel N, Heath AC, Larson-Prior L, Marcus D, Michalareas G, Moeller S, Oostenveld R, et al (2012) The Human Connectome Project: a data acquisition perspective. Neuroimage 62:2222–2231. 10.1016/j.neuroimage.2012.02.018 22366334 PMC3606888

[B70] Vidaurre D, Smith SM, Woolrich MW (2017) Brain network dynamics are hierarchically organized in time. Proc Natl Acad Sci U S A 114: 12827–12832. 10.1073/pnas.1705120114 29087305 PMC5715736

[B71] Vincent JL, Kahn I, Snyder AZ, Raichle ME, Buckner RL (2008) Evidence for a frontoparietal control system revealed by intrinsic functional connectivity. J Neurophysiol 100:3328–3342. 10.1152/jn.90355.2008 18799601 PMC2604839

[B72] Wen X, Rangarajan G, Ding M (2013) Is Granger causality a viable technique for analyzing fMRI data? PLoS One 8:e67428 10.1371/journal.pone.0067428 23861763 PMC3701552

[B73] Wilcox RR (1994) The percentage bend correlation coefficient. Psychometrika 59:601–616. 10.1007/BF02294395

