# Supplementary material for: Decoding Task-Specific Cognitive States with Slow, Directed Functional Networks in the Human Brain
Source: eNeuro. 2020 Jul 7;7(4):ENEURO.0512-19.2019. doi: 10.1523/ENEURO.0512-19.2019 (PMC7358332; doi:10.1523/ENEURO.0512-19.2019)
Supplement: Figure 1-1 — Task descriptions. Description of fMRI scans and tasks used in the analysis. Download Figure 1-1, DOC file. [file enu-eN-TNC-0512-19-s01.doc]

**Extended Data Figure 1-1. Task descriptions.**

Description of fMRI scans and tasks used in the analysis.

| Task Name/Key | Description | # Volumes | Time  (mm:ss) | # Runs | # Subjects |
| --- | --- | --- | --- | --- | --- |
| **Emotion Processing (E)** | Valence judgements (faces) and shape recognition | 176 | 02:16 | 2 | 1000 |
| **Gambling (G)** | Reward, punishment, decision making | 253 | 03:12 | 2 | 1000 |
| **Language (L)** | Sentences, stories, mental arithmetic(auditory) | 316 | 03:57 | 2 | 1000 |
| **Motor (M)** | Hand, foot, tongue movements | 284 | 03:34 | 2 | 1000 |
| **Relational Processing (R)** | Higher-order cognition | 232 | 02:56 | 2 | 991 |
| **Social Cognition (S)** | Interpret social vs. random interaction | 274 | 03:27 | 2 | 1000 |
| **Working Memory (W)** | N-back working memory, body parts, tools, places | 405 | 05:01 | 2 | 1000 |
| **Resting state** | Resting state with eyes open, relaxed fixation. | 1200 | 14:24 | 2 | 1000 |
