# Supplementary material for: Decoding Task-Specific Cognitive States with Slow, Directed Functional Networks in the Human Brain
Source: eNeuro. 2020 Jul 7;7(4):ENEURO.0512-19.2019. doi: 10.1523/ENEURO.0512-19.2019 (PMC7358332; doi:10.1523/ENEURO.0512-19.2019)
Supplement: Figure 1-2 — Description of subtasks. Download Figure 1-2, DOC file. [file enu-eN-TNC-0512-19-s02.doc]

**Extended Data Figure 1-2. Description of sub-tasks**

| Task Name | Sub-tasks and descriptions | # Time points |
| --- | --- | --- |
| **Emotion Processing** | Fear: emotional face matching blocks  Neutral: shape matching blocks | 67 |
| **Gambling** | Win: blocks with mostly reward  Loss: blocks with mostly loss | 80 |
| **Language** | Math: blocks for solving math problems  Story: blocks with story-based questions | 135 |
| **Motor** | Hand: left, right finger movement blocks  Foot: left, right toe movement blocks | 72 |
| **Relational Processing** | Relation: relational processing blocks  Match: shape and texture matching blocks | 72 |
| **Working Memory** | 0bk: 0-back working memory blocks  2bk: 2-back working memory blocks | 159 |
