# Supplementary material for: Decoding Task-Specific Cognitive States with Slow, Directed Functional Networks in the Human Brain
Source: eNeuro. 2020 Jul 7;7(4):ENEURO.0512-19.2019. doi: 10.1523/ENEURO.0512-19.2019 (PMC7358332; doi:10.1523/ENEURO.0512-19.2019)
Supplement: Figure 1-3 — Subject identifiers. HCP IDs of 1000 subjects whose data were used in the analysis. Relational processing scans were not available for IDs marked in gray. Download Figure 1-3, DOC file. [file enu-eN-TNC-0512-19-s03.doc]

**Extended Data Figure 1-3. Subject identifiers.**

HCP IDs of 1000 subjects whose data was employed in the analysis. Relational processing scans were not available for IDs marked in grey.

| **100206** | **111211** | **121921** | **133019** | **144933** | **154431** | **165941** | **177645** | **191841** | **202820** | **213522** | **287248** | **360030** | **436845** | **540436** | **592455** | **677968** | **763557** | **849264** | **917558** |
| --- | --- | --- | --- | --- | --- | --- | --- | --- | --- | --- | --- | --- | --- | --- | --- | --- | --- | --- | --- |
| **100307** | **111312** | **122317** | **133625** | **145127** | **154532** | **166438** | **178142** | **191942** | **203418** | **214019** | **290136** | **361234** | **441939** | **541640** | **594156** | **679568** | **765056** | **849971** | **919966** |
| **100408** | **111413** | **122418** | **133827** | **145632** | **154734** | **166640** | **178243** | **192035** | **203923** | **214221** | **293748** | **361941** | **445543** | **541943** | **597869** | **679770** | **765864** | **852455** | **922854** |
| **100610** | **111514** | **122620** | **133928** | **145834** | **154835** | **167036** | **178647** | **192136** | **204016** | **214423** | **295146** | **365343** | **448347** | **545345** | **598568** | **680250** | **767464** | **856463** | **923755** |
| **101006** | **111716** | **122822** | **134021** | **146129** | **154936** | **167238** | **178748** | **192237** | **204218** | **214524** | **297655** | **366042** | **449753** | **548250** | **599065** | **680452** | **769064** | **856766** | **926862** |
| **101107** | **112112** | **123117** | **134223** | **146331** | **155231** | **167440** | **178849** | **192439** | **204319** | **214726** | **298051** | **366446** | **453441** | **550439** | **599469** | **680957** | **770352** | **856968** | **927359** |
| **101309** | **112314** | **123420** | **134324** | **146432** | **155635** | **167743** | **178950** | **192540** | **204420** | **217126** | **298455** | **368753** | **453542** | **552241** | **599671** | **683256** | **771354** | **857263** | **930449** |
| **101410** | **112516** | **123521** | **134425** | **146533** | **155938** | **168139** | **179245** | **192641** | **204521** | **217429** | **299154** | **371843** | **454140** | **552544** | **601127** | **686969** | **773257** | **859671** | **932554** |
| **101915** | **112920** | **123723** | **134728** | **146735** | **156031** | **168240** | **179346** | **192843** | **204622** | **219231** | **299760** | **376247** | **456346** | **553344** | **604537** | **687163** | **774663** | **861456** | **933253** |
| **102008** | **113215** | **123824** | **134829** | **146836** | **156233** | **168341** | **180129** | **193239** | **205119** | **220721** | **300618** | **377451** | **459453** | **555348** | **609143** | **688569** | **779370** | **865363** | **942658** |
| **102109** | **113316** | **123925** | **135124** | **146937** | **156334** | **168745** | **180230** | **193845** | **205220** | **221319** | **300719** | **378756** | **461743** | **555651** | **611938** | **690152** | **782561** | **867468** | **943862** |
| **102311** | **113619** | **124220** | **135225** | **147030** | **156435** | **168947** | **180432** | **194140** | **205725** | **223929** | **303119** | **378857** | **463040** | **555954** | **613538** | **692964** | **783462** | **869472** | **947668** |
| **102513** | **113922** | **124422** | **135528** | **147636** | **156536** | **169040** | **180533** | **194443** | **205826** | **224022** | **303624** | **379657** | **465852** | **557857** | **614439** | **693764** | **784565** | **870861** | **951457** |
| **102614** | **114116** | **124624** | **135629** | **147737** | **156637** | **169444** | **180735** | **194645** | **206222** | **227432** | **304020** | **380036** | **467351** | **558657** | **615744** | **694362** | **786569** | **871762** | **952863** |
| **102715** | **114217** | **124826** | **135730** | **148032** | **157336** | **169545** | **180836** | **194746** | **206323** | **227533** | **304727** | **381038** | **468050** | **558960** | **616645** | **695768** | **788674** | **871964** | **955465** |
| **102816** | **114318** | **125222** | **135932** | **148133** | **157437** | **169747** | **180937** | **194847** | **206525** | **228434** | **305830** | **381543** | **469961** | **559053** | **617748** | **698168** | **788876** | **872562** | **957974** |
| **103111** | **114419** | **125424** | **136227** | **148335** | **157942** | **169949** | **181131** | **195041** | **206727** | **231928** | **307127** | **382242** | **473952** | **559457** | **618952** | **700634** | **789373** | **872764** | **958976** |
| **103212** | **114621** | **125525** | **136631** | **148436** | **158035** | **170631** | **181232** | **195445** | **206828** | **233326** | **308129** | **385046** | **475855** | **561242** | **620434** | **701535** | **792564** | **873968** | **959574** |
| **103414** | **114823** | **126325** | **136732** | **148840** | **158136** | **170934** | **181636** | **195849** | **206929** | **236130** | **308331** | **385450** | **479762** | **561444** | **622236** | **702133** | **792766** | **877269** | **962058** |
| **103515** | **114924** | **126426** | **136833** | **148941** | **158338** | **171330** | **182436** | **195950** | **207123** | **237334** | **309636** | **386250** | **480141** | **561949** | **623844** | **704238** | **792867** | **878776** | **965367** |
| **103818** | **115017** | **126628** | **137027** | **149236** | **158540** | **171532** | **182739** | **196144** | **207426** | **238033** | **310621** | **387959** | **481042** | **562345** | **626648** | **705341** | **793465** | **878877** | **965771** |
| **104012** | **115219** | **127226** | **137128** | **149337** | **158843** | **171633** | **183034** | **196346** | **208024** | **239136** | **311320** | **389357** | **481951** | **562446** | **627549** | **707749** | **800941** | **880157** | **966975** |
| **104416** | **115320** | **127327** | **137229** | **149539** | **159138** | **172029** | **183337** | **196750** | **208125** | **239944** | **314225** | **390645** | **485757** | **565452** | **627852** | **709551** | **802844** | **882161** | **969476** |
| **104820** | **115724** | **127630** | **137532** | **149741** | **159239** | **172130** | **183741** | **196851** | **208226** | **245333** | **316633** | **391748** | **486759** | **566454** | **628248** | **715041** | **803240** | **884064** | **970764** |
| **105014** | **115825** | **127832** | **137633** | **149842** | **159340** | **172332** | **185038** | **197348** | **208327** | **246133** | **316835** | **392447** | **492754** | **567052** | **633847** | **715950** | **809252** | **885975** | **971160** |
| **105115** | **116221** | **127933** | **137936** | **150524** | **159441** | **172433** | **185139** | **197550** | **209127** | **248339** | **317332** | **392750** | **495255** | **567759** | **634748** | **720337** | **810843** | **886674** | **972566** |
| **105216** | **116524** | **128026** | **138130** | **150625** | **159744** | **172534** | **185341** | **198047** | **209228** | **249947** | **318637** | **393247** | **497865** | **567961** | **635245** | **723141** | **812746** | **887373** | **973770** |
| **105620** | **116726** | **128127** | **138231** | **150726** | **159946** | **172938** | **185442** | **198249** | **209329** | **250427** | **320826** | **393550** | **499566** | **568963** | **638049** | **724446** | **814548** | **888678** | **978578** |
| **105923** | **117021** | **128632** | **138332** | **150928** | **160123** | **173334** | **185947** | **198350** | **209834** | **250932** | **321323** | **394956** | **500222** | **570243** | **644044** | **725751** | **814649** | **889579** | **979984** |
| **106016** | **117122** | **128935** | **138534** | **151021** | **160729** | **173435** | **186040** | **198451** | **209935** | **251833** | **322224** | **395251** | **506234** | **571144** | **644246** | **727553** | **815247** | **891667** | **983773** |
| **106319** | **117324** | **129028** | **138837** | **151223** | **160830** | **173536** | **186141** | **198653** | **210011** | **255639** | **325129** | **395756** | **510225** | **572045** | **645450** | **728454** | **816653** | **894067** | **984472** |
| **106521** | **117930** | **129129** | **139233** | **151324** | **161327** | **173637** | **186444** | **198855** | **210112** | **255740** | **329844** | **395958** | **510326** | **573249** | **645551** | **729557** | **818455** | **894673** | **987074** |
| **106824** | **118023** | **129331** | **139435** | **151425** | **161630** | **173738** | **186545** | **199150** | **210415** | **256540** | **330324** | **397154** | **512835** | **573451** | **647858** | **731140** | **818859** | **894774** | **987983** |
| **107018** | **118124** | **129634** | **139637** | **151526** | **161731** | **173839** | **186848** | **199352** | **210617** | **257542** | **333330** | **397760** | **513130** | **576255** | **654350** | **732243** | **820745** | **896778** | **989987** |
| **107321** | **118225** | **129937** | **139839** | **151627** | **161832** | **173940** | **187143** | **199453** | **211114** | **257845** | **334635** | **397861** | **513736** | **578057** | **654552** | **734045** | **825048** | **896879** | **990366** |
| **107422** | **118528** | **130013** | **140117** | **151728** | **162026** | **174437** | **187345** | **199655** | **211215** | **257946** | **336841** | **401422** | **516742** | **579665** | **654754** | **735148** | **825553** | **898176** | **991267** |
| **107725** | **118730** | **130114** | **140319** | **151829** | **162228** | **174841** | **187547** | **199958** | **211316** | **263436** | **339847** | **406432** | **517239** | **579867** | **656253** | **737960** | **825654** | **899885** | **992673** |
| **108020** | **118831** | **130316** | **140824** | **151930** | **162329** | **175035** | **187850** | **200008** | **211417** | **268749** | **341834** | **406836** | **518746** | **580044** | **656657** | **742549** | **826353** | **901038** | **992774** |
| **108121** | **118932** | **130417** | **140925** | **152225** | **162733** | **175136** | **188145** | **200109** | **211619** | **268850** | **342129** | **412528** | **519647** | **580650** | **657659** | **744553** | **826454** | **901139** | **993675** |
| **108222** | **119025** | **130518** | **141119** | **152427** | **162935** | **175237** | **188347** | **200210** | **211720** | **270332** | **346137** | **413934** | **519950** | **580751** | **660951** | **748258** | **828862** | **901442** | **994273** |
| **108323** | **119126** | **130619** | **141422** | **152831** | **163129** | **175338** | **188448** | **200311** | **211821** | **274542** | **346945** | **414229** | **520228** | **581349** | **662551** | **749058** | **832651** | **902242** | **996782** |
| **108525** | **119732** | **130720** | **141826** | **153025** | **163331** | **175439** | **188549** | **200513** | **211922** | **275645** | **348545** | **415837** | **522434** | **581450** | **663755** | **749361** | **833148** | **904044** | **137431** |
| **108828** | **119833** | **130821** | **142828** | **153126** | **163432** | **175742** | **188751** | **200614** | **212015** | **280739** | **349244** | **419239** | **523032** | **583858** | **664757** | **751348** | **833249** | **905147** | **143426** |
| **109123** | **120111** | **130922** | **143224** | **153227** | **163836** | **176037** | **189349** | **200917** | **212116** | **281135** | **350330** | **421226** | **524135** | **585256** | **665254** | **751550** | **835657** | **907656** | **169343** |
| **109325** | **120212** | **131217** | **143325** | **153429** | **164030** | **176239** | **189450** | **201111** | **212217** | **283543** | **352132** | **422632** | **525541** | **585862** | **667056** | **753150** | **837560** | **908860** | **182032** |
| **109830** | **120414** | **131419** | **143830** | **153631** | **164131** | **176441** | **189652** | **201414** | **212318** | **284646** | **352738** | **424939** | **529549** | **586460** | **671855** | **753251** | **837964** | **910241** | **199251** |
| **110007** | **120515** | **131722** | **144125** | **153833** | **164636** | **176542** | **190031** | **201515** | **212419** | **285345** | **353740** | **429040** | **529953** | **587664** | **672756** | **756055** | **841349** | **910443** | **289555** |
| **110411** | **120717** | **131823** | **144428** | **153934** | **164939** | **176845** | **191033** | **201818** | **212823** | **285446** | **355239** | **432332** | **530635** | **588565** | **673455** | **757764** | **843151** | **911849** | **615441** |
| **110613** | **121416** | **131924** | **144731** | **154229** | **165436** | **177140** | **191235** | **202113** | **213017** | **286347** | **356948** | **433839** | **531536** | **589567** | **675661** | **759869** | **844961** | **912447** | **715647** |
| **111009** | **121618** | **132017** | **144832** | **154330** | **165638** | **177241** | **191336** | **202719** | **213421** | **286650** | **358144** | **436239** | **536647** | **590047** | **677766** | **761957** | **845458** | **917255** | **729254** |
