# Supplementary material for: Decoding Task-Specific Cognitive States with Slow, Directed Functional Networks in the Human Brain
Source: eNeuro. 2020 Jul 7;7(4):ENEURO.0512-19.2019. doi: 10.1523/ENEURO.0512-19.2019 (PMC7358332; doi:10.1523/ENEURO.0512-19.2019)
Supplement: Figure 1-4 — Parcellations used in the analysis. Download Figure 1-4, DOC file. [file enu-eN-TNC-0512-19-s04.doc]

**Extended Data Figure 1-4. Parcellations used in the analysis.**

| Parcellation | Type | # Regions | Regions included |
| --- | --- | --- | --- |
| **AAL** | Anatomical | 116 | cortical  and subcortical |
| **Power** | Functional | 14 |  |
| **Shirer** |  | 90 |  |
| **Shirer (14)** |  | 14 |  |
| **Yeo** |  | 96 | cortical |
