# Supplementary material for: Decoding Task-Specific Cognitive States with Slow, Directed Functional Networks in the Human Brain
Source: eNeuro. 2020 Jul 7;7(4):ENEURO.0512-19.2019. doi: 10.1523/ENEURO.0512-19.2019 (PMC7358332; doi:10.1523/ENEURO.0512-19.2019)
Supplement: Figure 1-7 — Number of subjects passing stationarity tests. Download Figure 1-7, DOC file. [file enu-eN-TNC-0512-19-s07.doc]

**Extended Data Figure 1-7. Number of subjects passing stationarity tests.**

| Task | Stability test  (spectral radius) | Consistency test | Durbin-Watson whiteness test | Dicky-Fuller stationarity test | Common |
| --- | --- | --- | --- | --- | --- |
| **Rest** | 999 | 701 | 997 | 1000 | 697 |
| **Emotion** | 999 | 505 | 1000 | 1000 | 504 |
| **Gambling** | 1000 | 508 | 999 | 1000 | 507 |
| **Language** | 998 | 624 | 997 | 1000 | 621 |
| **Motor** | 1000 | 584 | 995 | 1000 | 580 |
| **Relational** | 991 | 717 | 989 | 991 | 716 |
| **Social** | 1000 | 681 | 997 | 1000 | 678 |
| **WM** | 1000 | 600 | 996 | 1000 | 597 |
