# Supplementary material for: Decoding Task-Specific Cognitive States with Slow, Directed Functional Networks in the Human Brain
Source: eNeuro. 2020 Jul 7;7(4):ENEURO.0512-19.2019. doi: 10.1523/ENEURO.0512-19.2019 (PMC7358332; doi:10.1523/ENEURO.0512-19.2019)
Supplement: Extended Data 1 — Mathematical Note. The MATLAB codes to reproduce the results are available at https://figshare.com/s/9d9131a6780fc8197cf1. Separate folders correspond to each figure, and subfolders contain scripts for generating each panel in the respective figure. The filenames are alphabetically ordered to provide a sequence for running the scripts. The Multivariate Granger Causality toolbox (mvgc_v1.0; available at http://users.sussex.ac.uk/~lionelb/downloads/mvgc_v1.0.zip) is a prerequisite. Data necessary to run the scripts (both input and output) are placed in a “data” subfolder within each figure folder. Download Extended Data 1, PDF file. [file enu-eN-TNC-0512-19-s14.pdf]

Extended Data: Mathematical Note

Decoding task-specific cognitive states with slow, directed functional networks in the human brain

S1 Granger-Geweke Causality estimation using MVAR modelling (Geweke, 1984)

Consider the multivariate random process $\mathbf{q}(t)$ whose time evolution is modeled by the following vector autoregressive model ("full" model):

$$\mathbf{q}(t) = \sum_{i=1}^{p} A_i \mathbf{q}(t-p) + \epsilon(t) \tag{1}$$

Let $\mathbf{q} = [\mathbf{x}^\top \mathbf{y}^\top \mathbf{z}^\top]^\top$, where $\mathbf{x}, \mathbf{y}$ and $\mathbf{z}$ are themselves multivariate random processes. $epsilon(t)$ is the residual (prediction error) of the full model, with covariance T. T can be expressed as comprising the following block matrices:

$$T = \begin{bmatrix} T_{\mathbf{x}} & T_{\mathbf{xy}} & T_{\mathbf{xz}} \\ T_{\mathbf{xy}}^\top & T_{\mathbf{y}} & T_{\mathbf{yz}} \\ T_{\mathbf{xz}}^\top & T_{\mathbf{yz}}^\top & T_{\mathbf{z}} \end{bmatrix} \tag{2}$$

where the submatrices $T_{\mathbf{x}}, T_{\mathbf{y}}$ and $T_{\mathbf{z}}$ are the covariances of the residuals associated with $\mathbf{x}, \mathbf{y}$ and $\mathbf{z}$ (rows), respectively, and $T_{\mathbf{xy}}$ etc are the covariance between the residuals associated with $\mathbf{x}$ and $\mathbf{y}$, etc.
We define the following *reduced* vector autoregressive processes.

$$\mathbf{x}(t) = \sum_{i=1}^{p} A_i^{(\mathbf{xx})} \mathbf{x}(t-p) + \sum_{i=1}^{p} A_i^{(\mathbf{xz})} \mathbf{z}(t-p) + \mathbf{u}(t) \tag{3}$$

$$\mathbf{y}(t) = \sum_{i=1}^{p} A_i^{(\mathbf{yy})} \mathbf{y}(t-p) + \sum_{i=1}^{p} A_i^{(\mathbf{yz})} \mathbf{z}(t-p) + \mathbf{v}(t) \tag{4}$$

where $\mathbf{u}$ and $\mathbf{v}$ are the residuals of the reduced VAR models, with respective covariance matrices $S_{\mathbf{x}}$ and $S_{\mathbf{y}}$.

The conditional linear dependence between $\mathbf{x}$ and $\mathbf{y}$, conditioned on $\mathbf{z}$, is given by the following equations.

$$\mathcal{F}_{\mathbf{x}\rightarrow\mathbf{y}|\mathbf{z}} = \ln\frac{|S_{\mathbf{y}}|}{|T_{\mathbf{y}}|} \tag{5}$$

$$\mathcal{F}_{\mathbf{y}\rightarrow\mathbf{x}|\mathbf{z}} = \ln\frac{|S_{\mathbf{x}}|}{|T_{\mathbf{x}}|} \tag{6}$$

$$\mathcal{F}_{\mathbf{x}\circ\mathbf{y}|\mathbf{z}} = \ln\frac{|T_{\mathbf{x}}|\,|T_{\mathbf{y}}|}{|T'|} \tag{7}$$

$$\mathcal{F}_{\mathbf{x},\mathbf{y}|\mathbf{z}} = \mathcal{F}_{\mathbf{x}\rightarrow\mathbf{y}} + \mathcal{F}_{\mathbf{y}\rightarrow\mathbf{x}} + \mathcal{F}_{\mathbf{x}\circ\mathbf{y}} \tag{8}$$

$$= \ln\frac{|S_{\mathbf{x}}|\,|S_{\mathbf{y}}|}{|T'|} \tag{9}$$

where T' is a sub-matrix of T comprising only the covariance components pertaining to $\mathbf{x}$ and $\mathbf{y}$ such that $T' = \begin{bmatrix} T_{\mathbf{x}} & T_{\mathbf{xy}} \\ T_{\mathbf{xy}}^{\top} & T_{\mathbf{y}} \end{bmatrix}$

S2 Analytic computation of instantaneous correlations

We consider the case of a two node network and find the analytic solution of correlations between the node time series. The dynamics of the network are simulted with vector Ornstein-Uhlenbeck process of the form:

$$\dot{\mathbf{x}} = A\,\mathbf{x} + \epsilon$$

where $\mathbf{x} = [x^1\ x^2]^{\top}$, representing the activity or state of the each node. Here, we assume ϵ to be a zero mean Gaussian white noise process with covariance $\Sigma = I_{2\times 2}$. The zero-lag covariance $\Gamma(0)$ of time series $\mathbf{x}$ satisfies the Lyapunov equation:

$$A\,\Gamma(0) + \Gamma(0)\,A^T + \Sigma = 0$$

Let $A = \begin{bmatrix} a & c \\ d & b \end{bmatrix}$ and $\Gamma(0) = \begin{bmatrix} \sigma_{11} & \sigma_{12} \\ \sigma_{12} & \sigma_{22} \end{bmatrix}$. With some simple algebra, it can be shown that σ_{12}, the zero-lag cross-covariance between x^1 and x^2 is given by:

$$\boldsymbol{\sigma_{12} = \frac{(ac+bd)}{2(ab-cd)(a+b)}} \tag{10}$$

In the specific case of each node having the same strength of self connections (same time constant of individual nodes), such that $b = a$, the zero-lag covariance reduces to $\sigma_{12} = (c+d)/4(a^2 - cd)$.

Therefore, this zero-lag covariance is a non linear function of $c+d$. Importantly, the covariance (and correlation) is 0 for the case of reciprocal excitatory-inhibitory connectivity, for which $c = -d$. This is the reason why partial correlations failed to capture reciprocal E-I interactions, as shown in Figure 3C (main text). An intuitive explanation for this phenomenon can be obtained by visualizing the covariation between the components of $\mathbf{x}$, for the special case when x^1 excites x^2, and x^2 inhibits x^1, which effectively produces no discernible pattern of correlations across the two components (ED Figure 3-2B).

S3 Relationship between instantaneous Granger-Geweke causality (iGC) and partial correlations (PC)

We derive here the relationship between conditional iGC and PC assuming a VAR process of order 1. For this derivation, we employ the following notation: x or α (lowercase letter or Greek symbol), to denote a scalar, $\tilde{x}$ or $\tilde{\alpha}$ (with $\sim$ above) to denote a vector, and A (italicized capital letter) to denote a matrix.

Consider a multivariate first order autoregressive process $\tilde{q}(t)$ comprised of M+2 component processes: $x(t)$, $y(t)$ and $\tilde{z}(t)$, the last comprising M processes. We write the AR model (p = 1) for this process as:

$$\begin{aligned}
x(t+1) &= \alpha_x^x \, x(t) + \alpha_y^x \, y(t) + \tilde{\alpha}_z^x \, \tilde{z}(t) + \epsilon_x(t) \\
y(t+1) &= \alpha_x^y \, x(t) + \alpha_y^y \, y(t) + \tilde{\alpha}_z^y \, \tilde{z}(t) + \epsilon_y(t) \\
\tilde{z}(t+1) &= \tilde{\alpha}_x^z \, x(t) + \tilde{\alpha}_y^z \, y(t) + A_z^z \, \tilde{z}(t) + \tilde{\epsilon}_z(t)
\end{aligned} \tag{11}$$

We rewrite these equations in more compact matrix notation.

$$\begin{bmatrix} x(t+1) \\ y(t+1) \end{bmatrix}_{2\times 1} = \begin{bmatrix} \alpha_x^x & \alpha_y^x & \tilde{\alpha}_z^x \\ \alpha_x^y & \alpha_y^y & \tilde{\alpha}_z^y \end{bmatrix}_{2\times(M+2)} \begin{bmatrix} x(t) \\ y(t) \\ \tilde{z}(t) \end{bmatrix}_{(M+2)\times 1} + \begin{bmatrix} \epsilon_x(t) \\ \epsilon_y(t) \end{bmatrix}_{2\times 1} \tag{12}$$

$$\tilde{z}(t+1)_{M\times 1} = \begin{bmatrix} \tilde{\alpha}_x^z & \tilde{\alpha}_y^z & A_z^z \end{bmatrix}_{M\times(M+2)} \begin{bmatrix} x(t) \\ y(t) \\ \tilde{z}(t) \end{bmatrix}_{(M+2)\times 1} + \tilde{\epsilon}_z(t)_{M\times 1} \tag{13}$$

We next write the equation describing partial correlations between x and y.

$$\begin{aligned}
x(t) &= \tilde{\beta}_z^x \, \tilde{z}(t) + k_x(t) \\
y(t) &= \tilde{\beta}_z^y \, \tilde{z}(t) + k_y(t)
\end{aligned} \tag{14}$$

Again, we rewrite these equations in compact matrix notation:

$$\begin{bmatrix} x(t) \\ y(t) \end{bmatrix}_{2\times 1} = \begin{bmatrix} \tilde{\beta}_z^x & \tilde{\beta}_z^y \end{bmatrix}_{2\times M} \tilde{z}(t)_{M\times 1} + \begin{bmatrix} k_x(t) \\ k_y(t) \end{bmatrix}_{2\times 1} \tag{15}$$

Substituting for $\tilde{z}(t+1)$ in (15) from (13):

$$\begin{bmatrix} x(t+1) \\ y(t+1) \end{bmatrix} = \begin{bmatrix} \tilde{\beta}_z^x & \tilde{\beta}_z^y \end{bmatrix} \left(\begin{bmatrix} \tilde{\alpha}_x^z & \tilde{\alpha}_y^z & A_z^z \end{bmatrix} \begin{bmatrix} x(t) \\ y(t) \\ \tilde{z}(t) \end{bmatrix} + \tilde{\epsilon}_z(t) \right) + \begin{bmatrix} k_x(t+1) \\ k_y(t+1) \end{bmatrix} \tag{16}$$

Equating the right hand sides of equations (12) and (16).

$$E \begin{bmatrix} x(t) \\ y(t) \\ \tilde{z}(t) \end{bmatrix} + \begin{bmatrix} \epsilon_x(t) \\ \epsilon_y(t) \end{bmatrix} = W \begin{bmatrix} x(t) \\ y(t) \\ \tilde{z}(t) \end{bmatrix} + B\ \tilde{\epsilon}_z(t) + \begin{bmatrix} k_x(t+1) \\ k_y(t+1) \end{bmatrix} \tag{17}$$

where,

$$E_{2\times(M+2)} = \begin{bmatrix} \alpha_x^x & \alpha_y^x & \tilde{\alpha}_z^x \\ \alpha_x^y & \alpha_y^y & \tilde{\alpha}_z^y \end{bmatrix}; \qquad W_{2\times(M+2)} = \begin{bmatrix} \tilde{\beta}_z^x & \tilde{\beta}_z^y \end{bmatrix} \begin{bmatrix} \tilde{\alpha}_x^z & \tilde{\alpha}_y^z & A_z^z \end{bmatrix}; \qquad B_{2\times M} = \begin{bmatrix} \tilde{\beta}_z^x & \tilde{\beta}_z^y \end{bmatrix}$$

Rearranging (17):

$$\begin{bmatrix} k_x(t+1) \\ k_y(t+1) \end{bmatrix} = \begin{bmatrix} E - W \end{bmatrix} \begin{bmatrix} x(t) \\ y(t) \\ \tilde{z}(t) \end{bmatrix} + B\ \tilde{\epsilon}_z(t) + \begin{bmatrix} \epsilon_x(t) \\ \epsilon_y(t) \end{bmatrix} \tag{18}$$

We rewrite this equation as:

$$\begin{bmatrix} k_x(t+1) \\ k_y(t+1) \end{bmatrix} = \Lambda \begin{bmatrix} x(t) \\ y(t) \\ \tilde{z}(t) \end{bmatrix} + \mathrm{B}' \begin{bmatrix} \epsilon_x(t) \\ \epsilon_y(t) \\ \tilde{\epsilon}_z(t) \end{bmatrix} \tag{19}$$

where, $\Lambda_{2\times(M+2)} = (E - W)$ and $B'_{2\times(M+2)} = \begin{bmatrix} I_{2\times 2} | B_{2\times M} \end{bmatrix}$.

Partial correlations depend on the (assumed, stationary) covariance between k_x and k_y (K) and iGC depends on the covariance between ϵ_x and ϵ_y (Y).

$$\left\langle \begin{bmatrix} k_x(t+1) \\ k_y(t+1) \end{bmatrix} \begin{bmatrix} k_x(t+1) & k_y(t+1) \end{bmatrix} \right\rangle_t = K = \begin{bmatrix} k_{xx} & k_{xy} \\ k_{yx} & k_{yy} \end{bmatrix} \tag{20}$$

$$\left\langle \begin{bmatrix} \epsilon_x(t+1) \\ \epsilon_y(t+1) \end{bmatrix} \begin{bmatrix} \epsilon_x(t+1) & \epsilon_y(t+1) \end{bmatrix} \right\rangle_t = Y = \begin{bmatrix} \epsilon_{xx} & \epsilon_{xy} \\ \epsilon_{yx} & \epsilon_{yy} \end{bmatrix} \tag{21}$$

Computing these covariances from equation (19), we obtain the following simplified form:

$$K = \Lambda C \Lambda^\top + B' \Omega B'^\top \tag{22}$$

where, $\Omega_{(M+2)\times(M+2)}$ is the covariance matrix of $\tilde{\epsilon}$ and $C_{(M+2)\times(M+2)}$ is the covariance matrix of $\tilde{q}(t)$.[1].

[1] The cross terms are zero because:

$$\left\langle \begin{bmatrix} x(t) \\ y(t) \\ \tilde{z}(t) \end{bmatrix} \begin{bmatrix} \epsilon_x(t) & \epsilon_y(t) & \tilde{\epsilon}_z(t) \end{bmatrix} \right\rangle_t = 0$$

We now make some simplifying assumptions by partitioning Ω as follows:

$$\Omega = \begin{bmatrix} Y & H \\ H^T & Z \end{bmatrix}$$

where, $Z_{M\times M} = \left\langle \tilde{\epsilon}_z, \tilde{\epsilon}_z^T \right\rangle_t$ and $H_{2\times M} = \left\langle \begin{bmatrix} \epsilon_x \\ \epsilon_y \end{bmatrix} \tilde{\epsilon}_z \right\rangle_t$. With the simplifying assumption that $H = 0_{2\times M}$, i.e., ϵ_x and ϵ_y are (separately) uncorrelated with components of $\tilde{\epsilon}_z$, equation (22) can be written as:

$$\boxed{\boldsymbol{K} = \Lambda \boldsymbol{C} \Lambda^\top + \boldsymbol{Y} + \boldsymbol{B}\boldsymbol{Z}\boldsymbol{B}^\top} \tag{23}$$

This shows that K (PC covariance) and Y (iGC covariance) are related but not identical. In fact, PC comprises contributions from both instantaneous connectivity (Y) and lagged connectivity (AR coefficients; Λ).

In the main text, ED Figure 3-2C, we demonstrate scenarios in which these quantities have distinct magnitudes, which lead to divergent predictions for PC and iGC functional connectivity estimates. It can be readily verified that removing lagged connectivity component, for example, by setting the MVAR coefficients to zero in equation (11), restores the identity between K and Y.
