# Supplementary material for: Decoding Task-Specific Cognitive States with Slow, Directed Functional Networks in the Human Brain
Source: eNeuro. 2020 Jul 7;7(4):ENEURO.0512-19.2019. doi: 10.1523/ENEURO.0512-19.2019 (PMC7358332; doi:10.1523/ENEURO.0512-19.2019)
Supplement: Figure 3-1 — Parameters of simulated networks. Parameters of two-node and nine-node networks. Download Figure 3-1, DOC file. [file enu-eN-TNC-0512-19-s08.doc]

**Extended Data Figure 3-1. Parameters of simulated networks.**

**A. 2-node network**

| Parameter | Symbol | Value (units) |
| --- | --- | --- |
| Number of neurons in each node | *N* | 1 |
| Single neuron timescale | τ | Network 1: 50 ms  Network 2: 1000 ms |
| Timestep for integration | Δ_t_ | 5 ms |
| Mean of residual noise | μ | 0 |
| Covariance of residual noise | Σ | Identity matrix |
| HRF scan repeat time | TR | 750 ms |
| Weight of inter-node connections |  | 1.5 |
| Number of repetitions |  | 25 |
| Number of simulated time points |  | 200 |
| GC/AR model order selection |  | AIC |

**B. 9-node network**

| Parameter | Symbol | Value (units) |
| --- | --- | --- |
| Number of neurons in each node | *N* | 100 |
| Probability of connections within each node | p | 0.1 |
| Single neuron timescale | τ | 50 ms |
| Variance of intra-node connections (w) | σ^2^_w_ | 4 |
| Mean of intra-node connections (w) | μ_w_ | Feedforward: 9.71  EE subnetwork:9.55  EE (balanced): 9.10  EI subnetwork: 9.77 |
| Timestep for integration | Δ_t_ | 5 ms |
| Mean of residual noise | μ | 0 |
| Covariance of residual noise | Σ | Identity matrix |
| Proportion of neurons with inter-node connections |  | 0.05 |
| Weight of inter-node connections (per connection) |  | Feedforward: 0.005  EE : 0.01, 0.0025  EE (balanced): 0.01  EI : 0.01, -0.01 |
| HRF scan repeat time | TR | 750 ms |
| Number of repetitions |  | 10 |
| Number of simulated time points |  | 200 |
| GC/AR model order selection |  | AIC |
