# Supplementary material for: Decoding Task-Specific Cognitive States with Slow, Directed Functional Networks in the Human Brain
Source: eNeuro. 2020 Jul 7;7(4):ENEURO.0512-19.2019. doi: 10.1523/ENEURO.0512-19.2019 (PMC7358332; doi:10.1523/ENEURO.0512-19.2019)
Supplement: Figure 4-1 — Network labels in the Shirer et al. (2012) 14-network parcellation. Download Figure 4-1, DOC file. [file enu-eN-TNC-0512-19-s10.doc]

**Extended Data Figure 4-1. Network labels in the Shirer et al (2012) 14-network parcellation**

| Region  ID | Abbreviated label | Description |
| --- | --- | --- |
| **1** | Aud | Auditory Network |
| **2** | BG | Basal Ganglia Network |
| **3** | l.ECN | Left DLPFC / Parietal (Left Executive Control Network) |
| **4** | Lang | Language Network |
| **5** | Prec | Precuneus Network |
| **6** | r.ECN | Right DLPFC / Parietal (Right Executive Control Network) |
| **7** | Sen.Mot | Sensorimotor Network |
| **8** | Vis.Spa | Intraparietal Sulcus / Frontal Eye Fields (Visuospatial Network) |
| **9** | a.Sal | Anterior Insula / Dorsal ACC (Anterior Salience Network) |
| **10** | d.DMN | PCC / MPFC (Dorsal Default Mode Network) |
| **11** | hi.Vis | Higher Visual Network |
| **12** | p.Sal | Posterior Insula (Posterior Salience Network) |
| **13** | pr.Vis | Primary Visual Network |
| **14** | v.DMN | Retrosplenial Cortex / Medial Temporal Lobe (Ventral Default Mode Network) |
