# Supplementary material for: Decoding Task-Specific Cognitive States with Slow, Directed Functional Networks in the Human Brain
Source: eNeuro. 2020 Jul 7;7(4):ENEURO.0512-19.2019. doi: 10.1523/ENEURO.0512-19.2019 (PMC7358332; doi:10.1523/ENEURO.0512-19.2019)
Supplement: Figure 5-1 — Behavioral scores and descriptions. Download Figure 5-1, DOC file. [file enu-eN-TNC-0512-19-s12.doc]

**Extended Data Figure 5-1. Behavioral scores and descriptions.**

| Index | Abbreviation | Age: adj/unadj | Description |
| --- | --- | --- | --- |
| **1** | MMSE_Score | - | Mini Mental Status Exam Total Score |
| **2** | PSQI_Score | - | Pittsburgh Sleep Questionnaire Total Score |
| **3** | Endurance | Adjusted | NIH Toolbox 2-minute Walk Endurance Test Score |
| **4** | Strength | Adjusted | NIH Toolbox Grip Strength Test Score |
| **5** | GaitSpeed_Comp | - | NIH Toolbox 4-Meter Walk Gait Speed Test: Computed Score |
| **6** | Dexterity | Adjusted | NIH Toolbox 9-hole Pegboard Dexterity Test Score |
| **7** | PicSeq | Adjusted | NIH Toolbox Picture Sequence Memory Test Score |
| **8** | CardSort | Adjusted | NIH Toolbox Dimensional Change Card Sort Test Score |
| **9** | Flanker | Adjusted | NIH Toolbox Flanker Inhibitory Control and Attention Test Score |
| **10** | FluInt_CR | - | Penn Progressive Matrices: Number of Correct Responses |
| **11** | ReadEng | Adjusted | NIH Toolbox Oral Reading Recognition Test Score |
| **12** | PicVocab | Adjusted | NIH Toolbox Picture Vocabulary Test Score |
| **13** | ProcSpeed | Adjusted | NIH Toolbox Pattern Comparison Processing Speed Test Score |
| **14** | DDisc_200 | - | Delay Discounting: Area Under the Curve for Discounting of $200 |
| **15** | DDisc_40K | - | Delay Discounting: Area Under the Curve for Discounting of $40,000 |
| **16** | VSPLOT_TC | - | Variable Short Penn Line Orientation: Total Number Correct |
| **17** | VSPLOT_OFF | - | Variable Short Penn Line Orientation: Total Positions Off for All Trials |
| **18** | SCPT_SEN | - | Short Penn Continuous Performance Test: Sensitivity |
| **19** | SCPT_SPEC | - | Short Penn Continuous Performance Test: Specificity |
| **20** | IWRD_TOT | - | Penn Word Memory Test: Total Number of Correct Responses |
| **21** | ListSort | Adjusted | NIH Toolbox List Sorting Working Memory Test Score |
| **22** | FluInt_SI | - | Penn Progressive Matrices: Total Skipped Items |
| **23** | FluInt _RTCR | - | Penn Progressive Matrices: Median Reaction Time for Correct Responses |
| **24** | VSPLOT_CRTE | - | Variable Short Penn Line Orientation: Median Reaction Time Divided by Expected Number of Clicks for Correct Trials |
| **25** | SCPT_TPRT | - | Short Penn CPT Median Response Time for True Positive Responses |
| **26** | SCPT_LRNR | - | Short Penn Continuous Performance Test: Longest Run of Non-Responses |
| **27** | IWRD_RTC | - | Penn Word Memory Test: Median Reaction Time for Correct Responses |
| **28** | ER40_CR | - | Penn Emotion Recognition Test: Number of Correct Responses |
| **29** | ER40ANG | - | Penn Emotion Recognition Test: Number of Correct Anger Identifications |
| **30** | ER40FEAR | - | Penn Emotion Recognition Test: Number of Correct Fear Identifications |
| **31** | ER40HAP | - | Penn Emotion Recognition Test: Number of Correct Happy Identifications |
| **32** | ER40NOE | - | Penn Emotion Recognition Test: Number of Correct Neutral Identifications |
| **33** | ER40SAD | - | Penn Emotion Recognition Test: Number of Correct Sad Identifications |
| **34** | LifeSatisf | Unadjusted | NIH Toolbox General Life Satisfaction Survey Score |
| **35** | MeanPurp | Unadjusted | NIH Toolbox Meaning and Purpose Survey Score |
| **36** | PosAff | Unadjusted | NIH Toolbox Positive Affect Survey Score |
| **37** | Friendship | Unadjusted | NIH Toolbox Friendship Survey Score |
| **38** | EmotSupp | Unadjusted | NIH Toolbox Emotional Support Survey Score |
| **39** | InstrSup | Unadjusted | NIH Toolbox Instrumental Support Survey Score |
| **40** | SelfEff | Unadjusted | NIH Toolbox Self-Efficacy Survey Score |
| **41** | ER40_CRT | Unadjusted | Penn Emotion Recognition Test: Correct Responses Median Response Time |
| **42** | AngAffect | Unadjusted | NIH Toolbox Anger-Affect Survey Score |
| **43** | AngHostil | Unadjusted | NIH Toolbox Anger-Hostility Survey Score |
| **44** | AngAggr | Unadjusted | NIH Toolbox Anger-Physical Aggression Survey Score |
| **45** | FearAff | Unadjusted | NIH Toolbox Fear-Affect Survey Score |
| **46** | FearSomat | Unadjusted | NIH Toolbox Fear-Somatic Arousal Survey Score |
| **47** | Sadness | Unadjusted | NIH Toolbox Sadness Survey Score |
| **48** | Loneliness | Unadjusted | NIH Toolbox Loneliness Survey Score |
| **49** | PercHostil | Unadjusted | NIH Toolbox Perceived Hostility Survey Score |
| **50** | PercReject | Unadjusted | NIH Toolbox Perceived Rejection Survey Score |
| **51** | PercStress | Unadjusted | NIH Toolbox Perceived Stress Survey Score |
